# Multimodal machine learning for modeling infant head circumference, mothers’ milk composition, and their shared environment

**DOI:** 10.1038/s41598-024-52323-w

**Published:** 2024-02-05

**Authors:** Martin Becker, Kelsey Fehr, Stephanie Goguen, Kozeta Miliku, Catherine Field, Bianca Robertson, Chloe Yonemitsu, Lars Bode, Elinor Simons, Jean Marshall, Bassel Dawod, Piushkumar Mandhane, Stuart E. Turvey, Theo J. Moraes, Padmaja Subbarao, Natalie Rodriguez, Nima Aghaeepour, Meghan B. Azad

**Affiliations:** 1 International Milk Composition (IMiC) Consortium, Winnipeg, Canada; 2 Manitoba Interdisciplinary Lactation Centre (MILC), Winnipeg, Canada; 3 Children’s Hospital Research Institute of Manitoba, Winnipeg, Canada; 4 University of Manitoba, Winnipeg, R3E3P4 Canada; 5 University of Toronto, Toronto, M5S 1A8 Canada; 6 McMaster University, Hamilton, M5S 1A8 Canada; 7 University of Alberta, Edmonton, T6G 2E1 Canada; 8 Dalhousie University, Halifax, B3H4R2 Canada; 9 University of British Columbia and British Columbia Children’s Hospital, Vancouver, V5Z4H4 Canada; 10 Stanford University, Stanford, 94305 USA; 11 University of California, San Diego, La Jolla, CA 92093 USA; 12 SickKids, Toronto, M5G 0A4 Canada

**Keywords:** Machine learning, Paediatric research

## Abstract

Links between human milk (HM) and infant development are poorly understood and often focus on individual HM components. Here we apply multi-modal predictive machine learning to study HM and head circumference (a proxy for brain development) among 1022 mother-infant dyads of the CHILD Cohort. We integrated HM data (19 oligosaccharides, 28 fatty acids, 3 hormones, 28 chemokines) with maternal and infant demographic, health, dietary and home environment data. Head circumference was significantly predictable at 3 and 12 months. Two of the most associated features were HM n3-polyunsaturated fatty acid C22:6n3 (docosahexaenoic acid, DHA; p = 9.6e−05) and maternal intake of fish (p = 4.1e−03), a key dietary source of DHA with established relationships to brain function. Thus, using a systems biology approach, we identified meaningful relationships between HM and brain development, which validates our statistical approach, gives credence to the novel associations we observed, and sets the foundation for further research with additional cohorts and HM analytes.

## Introduction

Physical growth and physiological development are complex processes influenced by a wide variety of factors across different domains (i.e., modalities), especially during the critical periods of gestation and infancy. These modalities include maternal dietary intake during pregnancy and/or lactation^1–3^, sociodemographic characteristics (e.g., age, socioeconomic status)^4^, the home environment (e.g., smokers, pets, cleaning chemicals)^5^, infant morbidities (e.g., diarrhea)^6^, infant feeding patterns (e.g., breastfeeding consistency)^7^, as well as human milk composition^8^. Human milk (HM) is a particularly complex factor as it comprises thousands of nutritive and non-nutritive components that collectively support infant growth and development and are, in turn, influenced by many of the above-mentioned modalities. Analyzing and modeling human milk as a biological system that fundamentally connects mother-infant dyads is a challenging but essential task that will lead to a better understanding of healthy infant growth and help to prevent developmental disorders^9–11^.

In this context, many studies have linked individual factors or modalities to infant growth and development—including head circumference (reflecting both physical growth and brain development^12,13^) and cognitive or behavioral outcomes (such as the Bayley Scales of Infant and Toddler Development^14^ or the Ages and Stages Questionnaire^15^). For example, *maternal dietary intake* during lactation can directly influence the nutritional composition of HM^16^ with long-term consequences for infants^1–3^. Specific to cognition, maternal fish consumption leads to higher docosahexaenoic acid (DHA) concentrations in HM, which in turn has been linked to infant brain development^17^ even if it is unclear whether this relationship influences infant growth in general^3,18^. *Maternal characteristics* such as age, body composition^12^, and socioeconomic status (e.g., maternal education or income) have been associated with infant growth and head circumference^4^. Similarly, maternal smoking habits can negatively influence infant growth^5,19^. *Home environment* factors like pets or cleaning chemicals can have an influence on infant development^20^. Additionally, many studies find that *infant morbidities* like diarrhea or pneumonia can impair infant growth^6,21^. Current evidence indicates that *infant feeding patterns* such as longer duration of exclusive or partial breastfeeding tended to be associated with healthier growth patterns during infancy (i.e., slower growth rate and earlier peak BMI in developed settings^7,22^)) and a reduced risk of overweight and obesity at ages 2 years and older^23^. Similarly, some but not all observational studies of term infants fed HM have reported enhanced brain development (through imaging studies), higher intelligence quotient scores and increased cognitive and behavioral outcomes (using validated scales) compared to formula-fed infants^8,24–28^, although this benefit has not been linked to any particular HM component.

Despite this large body of literature, the intricate relationships between the multitude of HM components and a plethora of maternal, infant, and environmental factors are poorly understood because research has typically focused on selected modalities and individual HM components or component types^42^. There is a growing recognition among researchers that multidisciplinary and systems biology approaches are required to decipher these complex relationships^9–11^. The International Milk Composition (IMiC) Consortium (www.milcresearch.com/imic) was established to address these knowledge gaps by collecting a wide variety of data modalities, including the measurement of an extensive array of HM components. However, analyzing such large amounts of data is analytically challenging and requires advanced statistical tools.

**Figure 1:**
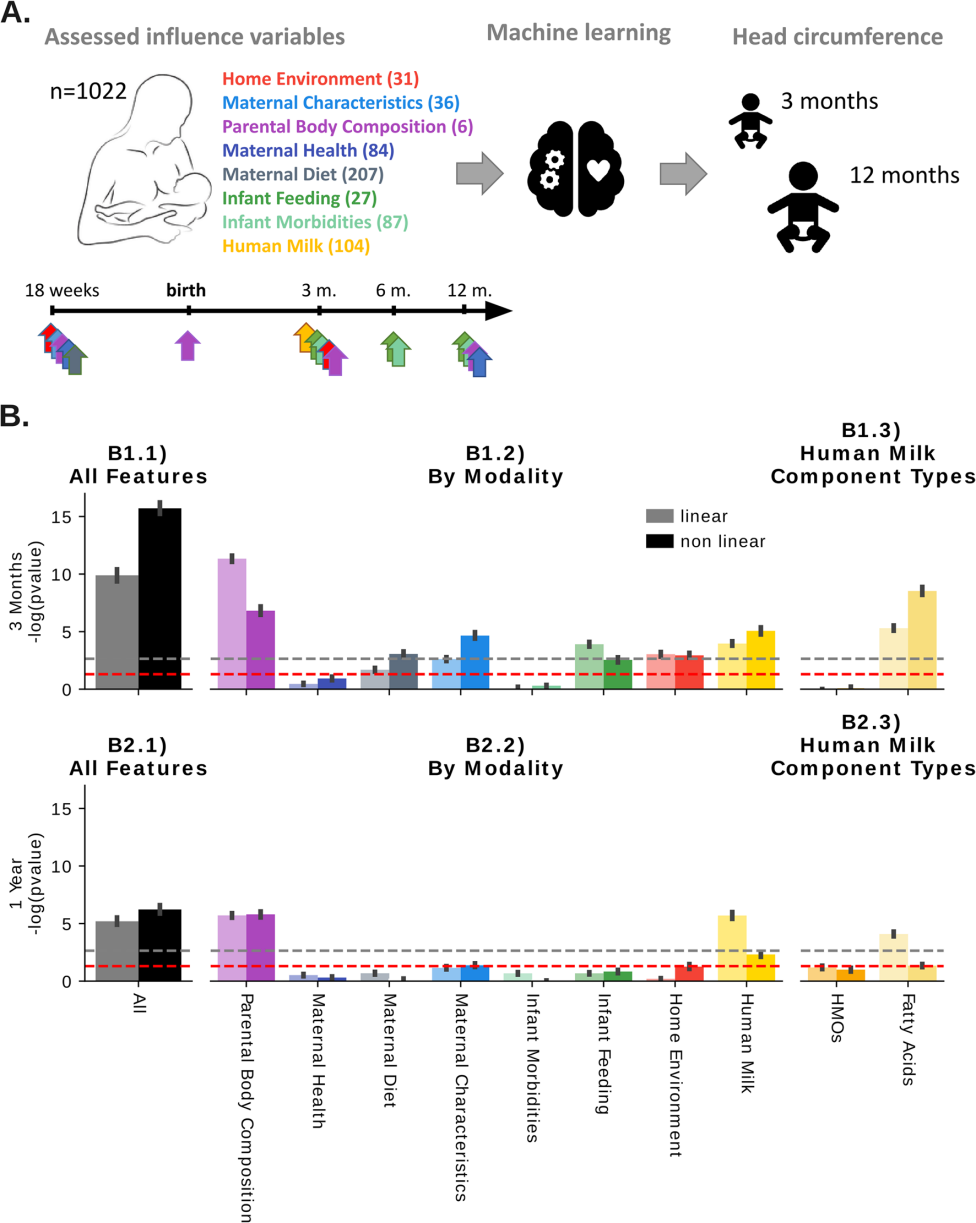
Study design and predictive modeling of head circumference at 3 months and 1 year using CHILD Cohort Study data (n = 1022 mother-infant dyads; n = 672 features. (**A**) A multitude of variables (features) across eight categories (modalities) were assessed at different time points before and after birth. This includes Home Environment (35 variables), Maternal Characteristics (42), Parental Body Composition (8), Maternal Health (149), Maternal Diet (207), Infant Feeding (45), Infant Morbidities (95), Human Milk (157). Using machine learning approaches, we then jointly integrate these data modalities to model infant head circumference (z-score for age) at 3 months and 1 year, respectively. For a full list of features, see Supplemental Table S1) Summarizes results from linear (ridge regression) and nonlinear (support vector machines) models using different combinations of features for prediction. Bars indicate the significance of the predictive power for each data subset and model measured by the negative log p-value of the association between the prediction and head circumference (Spearman), with 95% confidence intervals (black lines). For Spearman r values, see Supplemental Fig. S3. Dashed red lines denote p-value < 0.05 thresholds indicating the statistical significance of the predictions without multiple hypothesis comparison correction, the grey dashed lines denote a Bonferroni corrected statistical significance threshold assuming 22 experiments. Bar colors correspond to the modality color scheme in this figure. Combining all features into a multi-modal model (**B1.1**,**B2.1**) increases predictive power, particularly at 3 months. Predicting head circumference further into the future (1 year) is more challenging than short-term predictions (3 months). Human milk components are predictive (**B1.2**,**B2.2**), particularly fatty acids at 3 months (**B1.3**,**B2.3**). HMOs, human milk oligosaccharides. For a full list of features and modalities, see Supplemental Table S1.

Machine learning methods can simultaneously investigate a multitude of modalities and reveal clinically meaningful associations^29,30^. Here, we used machine learning to assess whether multiple data modalities, including HM composition, can predict current and/or future head circumference (a common proxy measure for brain development^31–34^) in breastfed infants. We integrated several existing datasets from the CHILD Cohort Study, a Canadian birth cohort with HM samples collected at 3 months. Specifically, HM data (oligosaccharides, fatty acids, cytokines, and hormones) were combined with maternal characteristics, diet, health and body composition, infant feeding and morbidities, as well as home environment information. This data was used to predict head circumference measured by study staff at 3 months and 1 year using machine learning (see Fig. 1 for an overview).

## Results

### Deep profiling of mother-infant dyads

The ongoing CHILD Cohort Study recruited 3624 pregnant Canadian women in 2009–2012 and has been tracking the growth and development of their children since birth. Additionally, a wide variety of data has been collected from periodic questionnaires, hospital records, as well as biological samples (see^35,36^ for more details). In the current study, we focus on a subcohort of 1022 CHILD mother-infant dyads with complete infant growth data through 1 year of age and available human milk data at 3–4 months (3 hormones measured by ELISA, 19 oligosaccharides measured by HPLC, 28 fatty acids measured by gas chromatography, and 28 immunomodulators including chemokines, cytokines, immunoglobulins and growth factors measured by Luminex assay or ELISA) (see “Materials and methods” for details). These data were combined with other modalities, including maternal characteristics (n = 36 features, e.g., age, and socioeconomic status), diet (n = 207, e.g., estimated nutrient and energy intakes, healthy eating index scores), body composition (n = 6, e.g., height, weight, BMI), and health (n = 84, e.g., depression, diabetes or asthma); infant morbidities (n = 87, e.g., diarrhea) and infant feeding (n = 27, e.g., breastfeeding duration and exclusivity); and home environment (n = 31, e.g., flooring and pets) (Supplementary Table S1).

As shown in Table 1, the mean age of mothers (n = 1022) was 32.99 (SD 4.23) years, and the mean infant age at milk collection was 3.73 (SD 1.07) months. Most mothers (91%) had a postsecondary degree, 27% were non-White, and 23% had asthma, while 34% were overweight (22%) or obese (12%). The median duration of exclusive breastfeeding in this subcohort was 4.5 months, the median duration of any breastfeeding was 1 year, and 77% of mothers reported feeding pumped milk to their infant before 3 months. Many houses had carpet installed (26%), and nearly half of all households had a pet (47%). Mean infant head circumference was 40.8 cm (SD 2.0) at 3 months (measured on the same date as HM sample collection) and 46.0 cm (SD 1.9) at 1 year. The Spearman rank correlation between the head circumference z-score at 3 months and 1 year was r = 0.37, p = 9.57E−35. Milk composition profiles have been described previously (separately) for human milk oligosaccharides (HMOs)^37^, fatty acids^38^, and hormones^39^.

**Table 1:** Key demographic characteristics of mother-infant dyads from the CHILD cohort study included in the current analysis (n = 1022).

	Features	Average	n (%)
Mother	Age (years)	33.0 ± 4.2	
	Height (cm)	165.1 ± 7.5	
	Weight (kg)	66.8 ± 14.8	
	BMI (kg/m^2^)	24.5 ± 5.0	
	Overweight (%)		223 (22.5)
	Obese (%)		123 (12.4)
	Post-secondary education (%)		848 (90.6)
	Race (%)		
	Asian		210 (17.5)
	White		862 (71.9)
	First nations		46 (3.8)
	Other		79 (6.6)
	Asthma (%)		240 (23.5)
Infant	Gestational age at delivery (weeks)	39.7 ± 1.2	
	Age at milk collection (months)	3.8 ± 1.1	
	Sex (% female)		543 (53.1)
	Fever in first 3 months (%)		207 (20.3)
	Cold with diarrhea in first 3 months (%)		7 (0.7)
	Head circumference at 3 months (cm)	40.8 ± 2.0	
	Head circumference at 1 year (cm)	46.0 ± 1.9	
	Head circumference at 3 months (z-score)	0.0 ± 1.6	
	Head circumference at 1 year (z-score)	0.3 ± 1.4	
Feeding	Breastfeeding duration (max 12 months)	12.0 [8.0, 12.0]	
	Exclusive breastfeeding duration (months)	4.5 [1.0, 5.0]	
	Used pump at 3 months (%)		790 (77.3)
Home	Carpet (%)		262 (25.6)
	Pet (%)		479 (46.9)

Values are n (%) or mean ± standard deviation or median [interquartile range]. By design, all dyads in this subset of the CHILD cohort breastfed for at least 3 months (the time of breast milk sample collection).

### An integrated multi-omics model for head circumference

To analyze whether the collective information contained in the multi-modal data can jointly predict infant head circumference z-score by age (a key measure of growth and development^12,13^), we generated machine learning models based on a fivefold cross-validation scheme. To account for a large number of involved variables while at the same time ensuring that more complex relationships between these variables are captured, we employed linear (ridge regression with nested parameter optimization) as well as non-linear models (support vector machines with radial basis function kernels), respectively. See “Materials and methods” for details.

Figure 1B shows a summary of model performances for predicting head circumference at 3 months (Panel B1) and 1 year (Panel B2) across the different data types and data modalities. Using all data modalities combined, infant head circumference was significantly predictable at 3 months (Panel B1.1, Spearman r = 0.25, p = 3.5e−16) as well as 1 year (Panel B2.1, r = 0.15, p = 1.3e−06). While Fig. 1B shows a comparisons of p-values, Supplementary Fig. S3 visualizes the corresponding Spearman rank correlation coefficients.

At 3 months (Panel B1), maternal body composition (r = 0.21, p = 5.1e−12) followed by human milk composition (r = 0.14, p = 1.0e−05) and parental characteristics (r = 0.13, p = 3.7e−05) have the highest predictive association with head circumference across all modalities (Panel B1.2). Other predictive modalities included maternal diet (r = 0.10, p = 9.3e−04), infant feeding (r = 0.11, p = 2.5e−04), as well as home environment (r = 0.11, p = 5.7e−04). Only maternal health (r = 0.05, p = 1.2e−01) and infant morbidities (r = 0.01, p = 5.6e−01) did not show significant predictive power for head circumference at 3 months. Of the milk composition modalities included in model building (B1.3) fatty acids were the most associated with head circumference (r = 0.18, p = 2.8e−09) while HMOs did not show a collectively significant predictive signal.

Head circumference was less predictable at 1 year (Panel B2) compared to 3 months. This is to be expected as predicting further into the future adds uncertainty due to the increasing variety of factors that influence infant development which may not, or only incompletely, have been captured. Nevertheless, the combination of all modalities is still significantly predictive of head circumference at 1 year (Panel B2.1, r = 0.15, p = 1.3e−06). Furthermore, parental body composition (Panel B2.2, r = 0.15, p = 1.4e−06), as well as milk composition, remain significantly associated with head circumference (Panel B2.2, r = 0.15, p = 2.6e−06), and milk fatty acids are again the most predictive (Panel B2.3, r = 0.12, p = 7.6e−05). In contrast to the 3-month predictions, maternal characteristics, maternal diet, infant feeding, and home environment modalities are not significantly associated with head circumference at 1 year.

Overall, only parental body compositions and milk components are consistently predictive at 3 months as well as after 1 year (see Fig. 1B).

### Individual associations of features with head circumference

**Figure 2:**
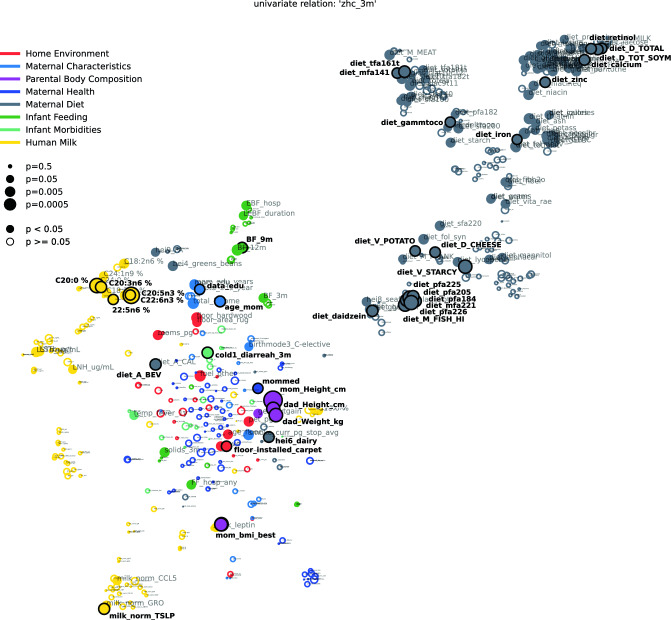
Feature interdependency network illustrating correlations among predictor variables and their associations with head circumference at 3 months. Each node corresponds to a feature from various modalities (encoded by color). The closer the features the more similar they can be considered with regard to their correlation structure. Node sizes represent the strength of association between head circumference at 3 months and the corresponding feature based on the p-value of a significance test (Kendall's Tau for continuous variables, Wilcoxon rank sum test for binary variables). If this association passes a significance threshold of p < 0.05, the corresponding feature is represented by a filled node (no multiple test correction for visualization purposes). Prominent clusters of human milk components (yellow), infant feeding (green), and maternal diet (grey) emerge. For a detailed description of features, see Supplemental Table S1.

**Figure 3:**
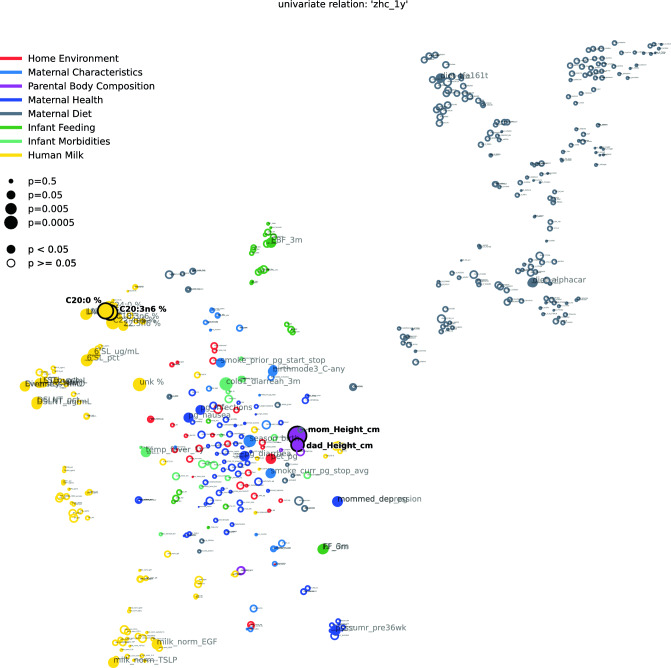
Feature interdependency network illustrating correlations among predictor variables and their associations with head circumference at 1 year. Analogously to Fig. 2, each node corresponds to a feature from various modalities (encoded by color). The closer the features the more similar they can be considered with regard to their correlation structure. Node sizes represent the strength of association between head circumference at twelve months and the corresponding feature based on the p-value of a respective significance test (Kendall's Tau for continuous variables, Wilcoxon rank sum test for binary variables). If this association passes a significance threshold of p < 0.05, the corresponding feature name is represented by a filled node (no multiple test correction for visualization purposes). Compared to head circumference at three months (Fig. 2), associations of features with head circumference at 1 year are much weaker (illustrated by fewer/smaller filled nodes). The remaining associations are concentrated in milk components. For a detailed description of features, see Supplemental Table S1.

**Figure 4:**
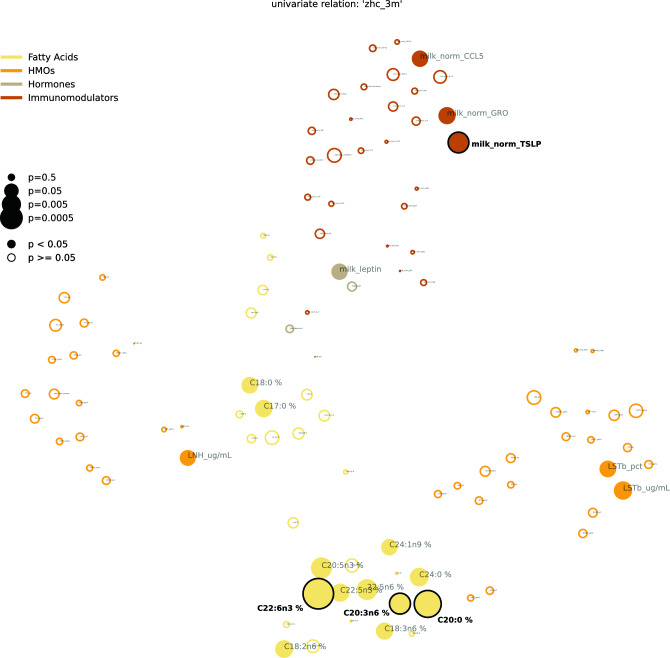
Feature interdependency network of human milk components illustrating correlations among predictor variables and their associations with head circumference at 3 months. Each node corresponds to a feature from various human milk modalities (encoded by color). The closer the features, the more similar they can be considered with regard to their correlation structure. Node sizes represent the strength of association between head circumference at 3 months and the corresponding feature based on the p-value of a respective significance test (Kendall's Tau for continuous variables, Wilcoxon rank sum test for binary variables). If this association passes a significance threshold of p < 0.05, the corresponding feature name is represented by a filled node (no multiple test correction for visualization purposes). Clusters emerge, grouping, for example, specific fatty acids and immunomodulators. For a detailed description of features, see Supplemental Table S1.

**Figure 5:**
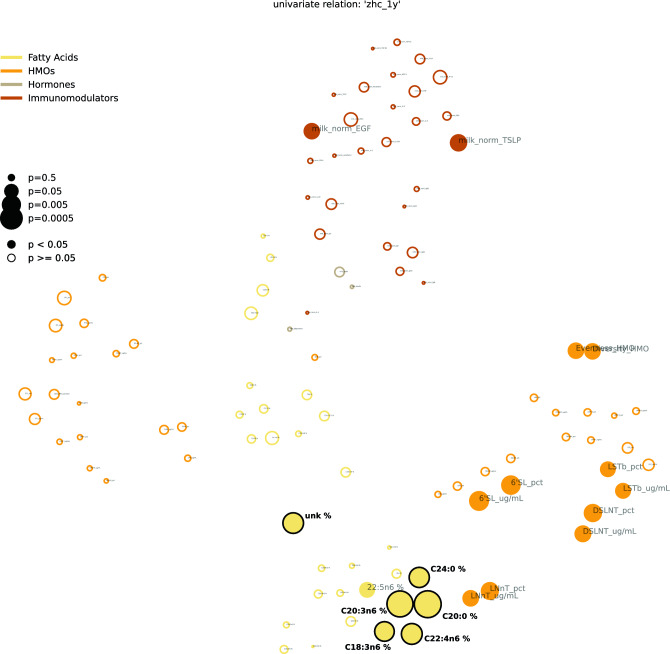
Feature interdependency network of human milk components illustrating correlations among predictor variables and their associations with head circumference at 1 year. Each node corresponds to a feature from various human milk modalities (encoded by color). The closer the features, the more similar they can be considered with regard to their correlation structure. Node sizes represent the strength of association between head circumference at 3 months and the corresponding feature based on the p-value of a respective significance test (Kendall's Tau for continuous variables, Wilcoxon rank sum test for binary variables). If this association passes a significance threshold of p < 0.05, the corresponding feature name is represented by a filled node (no multiple test correction for visualization purposes). Compared to associations at three months (Fig. 4), associations with head circumference appear increasingly in an HMO cluster. For a detailed description of features, see Supplemental Table S1.

We further investigated the relationship of individually measured variables and infant head circumference. To do this, we derived feature interdependency networks that visualize the correlation structure between variables while at the same time showing their association with head circumference at 3 months (Figs. 2, 4 and Tables 2, 4) and 1 year (Figs. 3, 5 and Tables 3, 5).

**Table 2:**
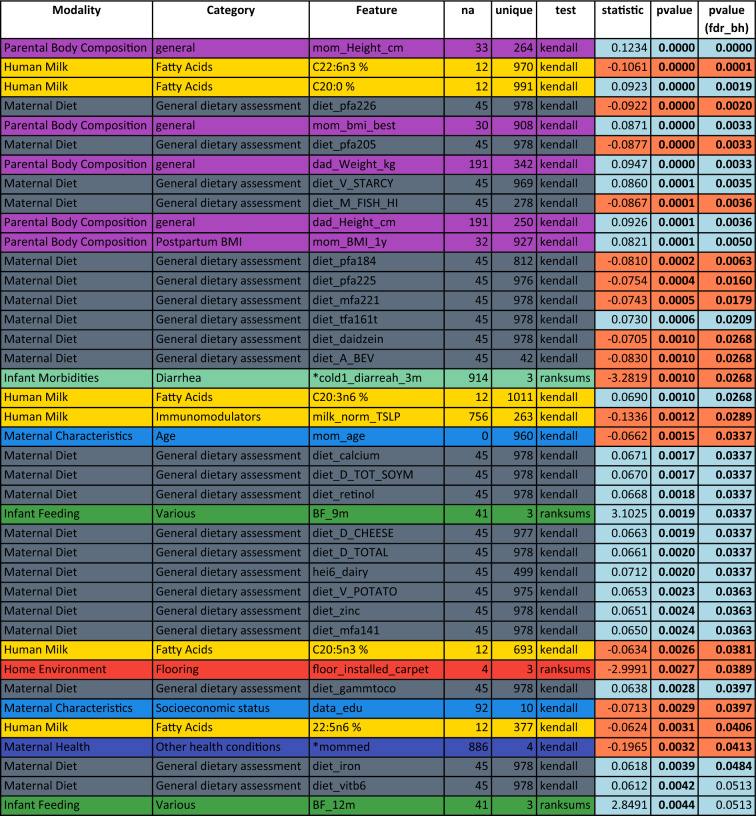
Univariate analysis of predictors of head circumference at 3 months in the CHILD cohort study: top 40 features sorted by p-value.

Associations are measured by Kendall’s Tau for numeric features or Wilcoxon rank sum for binary features. Feature colors correspond to modality types as in Fig. 1A. P-values in the last column are corrected using the Benjamini–Hochberg FDR procedure across all considered features. Significant features with and without correction are marked by bold font in the respective p-value column. A blue background in the p-value columns marks positive, and orange mark negative associations. A “*” indicate variables that depend on other variables (e.g., diarrhea is only assessed when the baby has a cold). The column “na” lists the number of missing values for the respective variable and “unique” denotes how many unique values the variable is represented by. For a detailed description of features, see Supplemental Table S1.

**Table 3:**
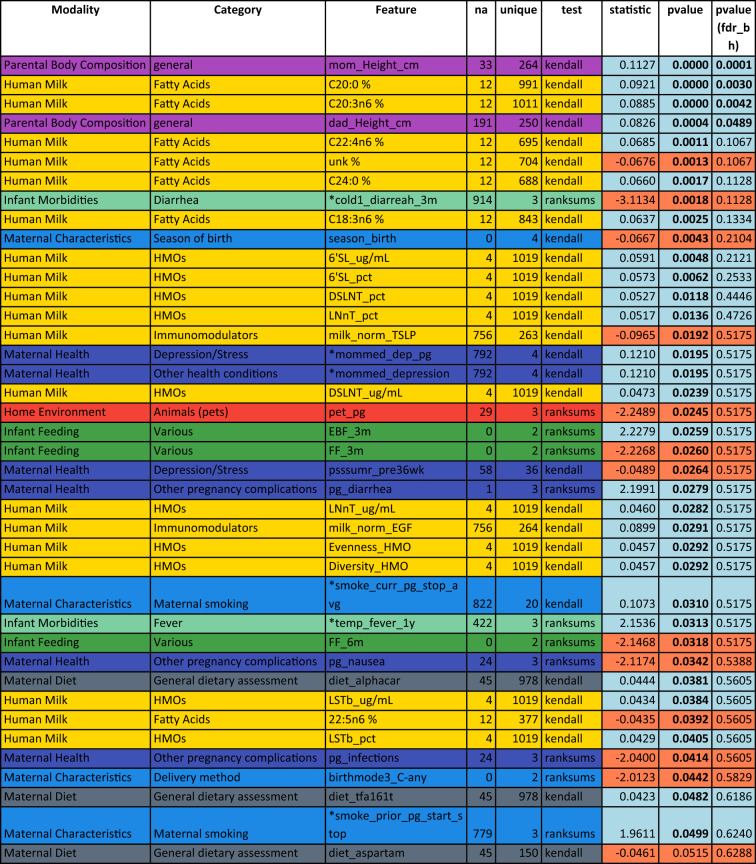
Univariate analysis of predictors of head circumference at 1 year in the CHILD cohort study: top 40 features sorted by p-value.

Associations are measured by Kendall’s Tau for numeric features or Wilcoxon rank sum for binary features. Feature colors correspond to modality types as in Fig. 1A. P-values in the last column are corrected using the Benjamini–Hochberg FDR procedure across all considered features. Significant features with and without correction are marked by bold font in the respective p-value column. A blue background in the p-value columns marks positive, and orange mark negative associations. A “*” indicate variables that depend on other variables (e.g., diarrhea is only assessed when the baby has a cold). The column “na” lists the number of missing values for the respective variable and “unique” denotes how many unique values the variable is represented by. For a detailed description of features, see Supplemental Table S1.

Figure 2 (all features) and Fig. 4 (HM components only) visualize the relationship between features (proximity of dots to each other) as well as their association with head circumference (size of dots) at 3 months. As expected, features of each modality tend to cluster together (e.g., maternal diet variables [grey cluster], HM components [several yellow clusters], infant feeding variables [green cluster]). Some features from different modalities are also clustered together in meaningful ways; for example, maternal BMI clustered with milk leptin and milk insulin levels, which are known to be strongly related to maternal body composition (Fig. 2). Among milk components, different modalities (e.g., fatty acids, HMOs, immunomodulators) tended to cluster separately, with sub-clusters emerging in some cases. For example, among milk fatty acids, the -n3 and -n6 polyunsaturated fatty acids clustered separately from the saturated fatty acids (CXX:0). Additionally, two distinct HMO clusters emerged, with one comprising HMOs strongly dependent on maternal secretor status (e.g. 2’FL, LNFP1, DFLac, DFLNT) and the other comprising HMOs relatively unrelated to secretor status (e.g. LNnT, DSLNT, 6’SL). The correlation networks also support and expand upon the predictive modeling results (Fig. 1B) by illustrating that many more features are associated with infant head circumference at 3 months (Fig. 2; many large filled circles) than at 1 year (Fig. 3, few large filled circles). At the same time, they give an overview of the association strength between each feature and head circumference at 3 months and 1 year, respectively.

Table 2 lists the top variables associated with head circumference at 3 months in univariate analyses (filled circles in Fig. 2). Notably, besides parental body composition-related variables (height and BMI), the single most associated feature to head circumference after 3 months was the n3-polyunsaturated fatty acid C22:6n3 (docosahexaenoic acid, DHA; p = 9.6e−05), which is widely known to influence infant brain development^17^. In addition, the commonly investigated polyunsaturated fatty acid metrics DHA + EPA (p = 2.7e−4, where EPA represents eicosapentaenoic acid, i.e., C20:5n3) as well DHA/ARA (p = 2.7e−4, where ARA represents arachidonic acid, i.e., C20:4n6) were significantly associated with head circumference at 3 months (not shown in table). However, contrasting previously found connections between maternal fish oil or DHA supplementation and infant head circumference^40,41^, we found increased DHA in HM to be associated with lower head circumference at 3 months (tau = − 0.11, p = 9.6e−05), pointing towards a negative relationship. The same is true for maternal fish intake (tau = − 0.08, p = 4.1e−03), which is a key dietary source of DHA. Other significantly associated features (all p < 3.0e−02) observed in our analysis included the HM saturated fatty acid C20:0 (positive association), various estimated PUFA intakes from FFQ data (negative), maternal height and body mass index (positive), infant diarrhea (negative), and maternal intake of alcohol (A_bev; negative) and starchy vegetables (M_STARCY; positive). See Supplementary Fig. S1 for a visualization of relationships between selected variables and head circumference at 3 months.

By 1 year (Fig. 3, Table 3), many of the feature associations with head circumference observed at 3 months (represented by filled circles in Fig. 2) were no longer evident. The strongest associations to head circumference at 1 year were primarily among human milk components, although interestingly, these were mostly different from the components associated with head circumference at 3 months. Notably, only two fatty acids (C20:0 and C20:3n6) are significantly associated with head circumference at both 3 months and 1 year (see Supplementary Fig. S2 for a visualization of C20:0). Additionally, HMOs are more prominent among the features significantly associated with head circumference at 1 year (without correction for multiple hypothesis comparison) compared at 3 month (cf. Tables 4 and 5).

**Table 4:**
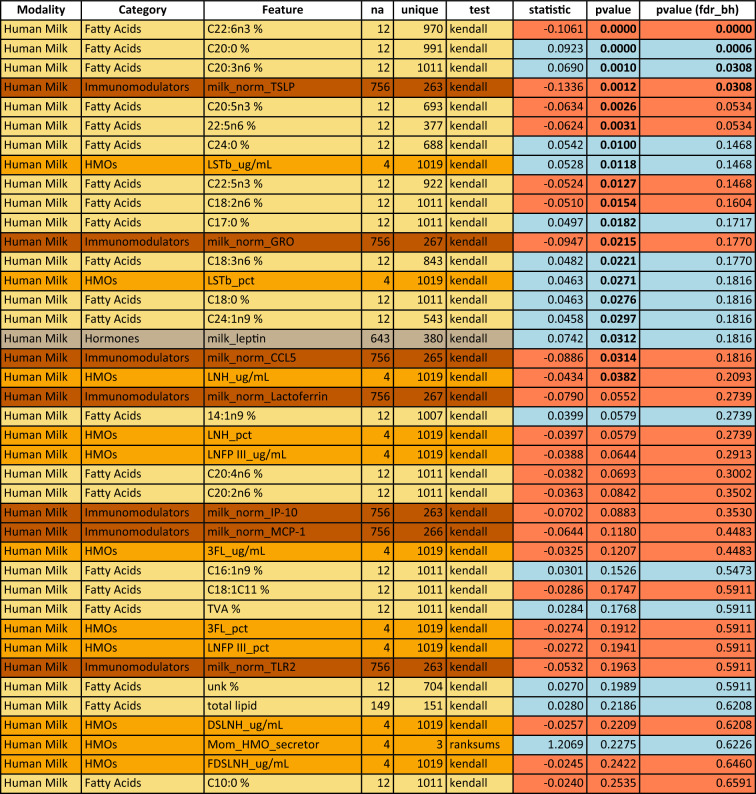
Univariate analysis of human milk predictors of head circumference at 3 months in the CHILD cohort study: top 40 human milk components sorted by p-value.

Associations are measured by Kendall’s Tau for numeric features or Wilcoxon rank sum for binary features. Feature colors correspond to modality types as in Fig. 4. P-values in the last column are corrected using the Benjamini–Hochberg FDR procedure across all considered features. Significant features with and without correction are marked by bold font in the respective p-value column. A blue background in the p-value columns marks positive, and orange mark negative associations. The column “na” lists the number of missing values for the respective variable and “unique” denotes how many unique values the variable is represented by. For a detailed description of features, see Supplemental Table S1.

**Table 5:**
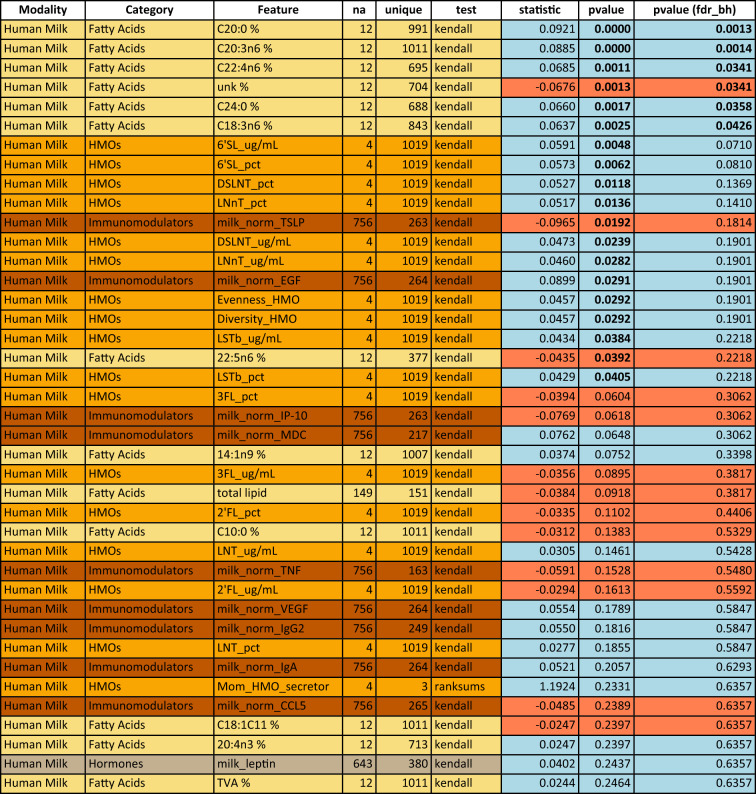
Univariate analysis of human milk predictors of head circumference at 1 year in the CHILD cohort study: top 40 human milk components sorted by p-value.

Associations are measured by Kendall’s Tau for numeric features or Wilcoxon rank sum for binary features. Feature colors correspond to modality types as in Fig. 5. P-values in the last column are corrected using the Benjamini–Hochberg FDR procedure across all considered features. Significant features with and without correction are marked by bold font in the respective p-value column. A blue background in the p-value columns marks positive, and orange mark negative associations. The column “na” lists the number of missing values for the respective variable and “unique” denotes how many unique values the variable is represented by. For a detailed description of features, see Supplemental Table S1.

Overall, the decreased association strengths of individual features with head circumference at 1 year could be explained by the more complex relationship of influence factors and developmental processes of infants as time progresses after pregnancy, which is also reflected by the multivariable modeling results discussed in the previous section and visualized in Fig. 1B where power decreases for the models at 1 year**.**

## Discussion

In this study of over 1000 mother-infant dyads, we applied a unique systems biology approach to model the complex relationships between infant head circumference (a proxy for brain development) and hundreds of maternal, infant, environmental, and HM factors. Among others, we identified a well-established pathway with potential practical impact (i.e., maternal fish intake elevates HM DHA, which impacts infant brain development—albeit in an unexpected direction), which validates our statistical approach, gives credence to the additional novel associations we observed, and sets the foundation for further analyses with additional dyads, cohorts and HM analytes.

### Integrated models increase predictive power and highlight the complexity of infant growth

Infant growth has been studied intensively in relation to many different factors, including feeding practices and HM components^42^. However, few, if any, have taken a systems biology approach. In this study, we jointly analyzed many different HM components together with other modalities in an integrated multivariate predictive model. This allowed us to simultaneously consider all included factors; an approach that has shown to be effective with regard to predictive power in other research areas. For example, integrating multiple modalities and omics has been successfully applied to model pregnancy progression^43^, predicting onset of labor^44^ and preeclampsia^45–47^. Our current findings highlight the complexity of infant growth and brain development and emphasize the need for further studies in diverse populations accounting for additional HM components and more sociodemographic factors and their interplay.

### Key predictors of infant head circumference

Consistent with previous research^48^, we found that maternal height was significantly associated with infant head circumference. After maternal height, the most associated feature to head circumference at 3 months was the HM n3-polyunsaturated fatty acid C22:6n3 (docosahexaenoic acid, DHA; p = 9.6e−05), which has a well-established role in supporting brain development. DHA is the most abundant omega-3 fatty acid in the brain and is implicated in several neuronal functions, including neurogenesis and neurotransmission^17,49,50^, although postnatal DHA supplementation trials in preterm^51^ and term^52^ infants have not shown consistent benefits for neurodevelopment, perhaps because DHA is mainly accumulated by the fetus during the last trimester of pregnancy^17^. Notably, in our study, increased DHA in HM seemed to be associated with *lower* head circumference at 3 months (tau = − 0.11, p = 9.6e−05), suggesting a potentially negative relationship. However, it is important to note that this finding is based on relative DHA proportions (not absolute concentrations), since information on total milk fat, calories, and volume was not available and therefore we could not determine the total “dose” of milk fatty acids delivered to each infant^38^. Further investigation is needed to confirm and understand the unexpected direction of this relationship.

Other features significantly associated with infant head circumference in our analysis included other HM fatty acids (e.g., C20:0, positive relationship), various estimated PUFA intakes from maternal FFQ data (negative), and maternal intake of alcohol (negative), fish (negative) and starchy vegetables (positive). This supports evidence that maternal diet during pregnancy and/or lactation can influence fetal/infant brain development^53–55^. Infant colds with diarrhea were associated with smaller head circumference at 3 months, suggesting that gut health and intestinal infections in early life could influence brain development.

### Complexity of modeling growth factors over time

The complexity of the different factors and data modalities and their relationship to infant growth is further highlighted by the reduced predictive power for head circumference after 1 year. Only parental body composition and milk components are consistently predictive at 3 months as well as after 1 year (see Fig. 1B), however, they still decrease in predictive power. This shows that predicting further into the future (1 year vs. 3 months) can be challenging and may require additional information or data to make accurate predictions or more powerful models. This also points towards external variables and influencing factors that continuously change and may require additional longitudinal monitoring of key variables during the growth period (for example, HM composition changes over time, but was only measured once in this study). In addition, for HM data in general and HM fatty acids in particular, we observed that non-linear models performed better at 3 months while linear models were superior at 1 year, hinting at more complex functional relationships that are picked up by these models at 3 months. As well, while HMOs did not predict head circumference at 3 months, they approached significance as predictors of head circumference at 1 year, suggesting that different HM components (in this case, fatty acids vs. HMOs) may contribute differentially to head growth or brain development at different stages of infancy, thus emphasizing the importance of a) broadly considering many HM components and b) examining child outcomes longitudinally.

### Limitations and future work

This proof-of-concept analysis is a starting point to explore the complex relationships among different predictors of HM composition and infant growth. For example, the microbiome of HM^56^ could actively modify (metabolize or synthesize) other HM components and directly or indirectly influence infant growth. Studying these intricate functional relationships may allow for more powerful predictive models and a deeper understanding of the underlying processes^57^. A limitation of our study is that some data were available for only a subset of dyads (e.g., immunomodulator and hormone data were only available for roughly 25% of dyads), and some key HM components were not analyzed (e.g., growth factors, micronutrients, and macronutrients). This is because we used an existing dataset that was assembled primarily to study associations with immune development and allergic disease, where nutrients were not prioritized. These gaps will be addressed in future studies within the International Milk Composition (IMiC) Consortium (www.milcresearch.com/imic), established specifically to study the complex associations between HM and infant growth. It is also important to note that, while significant, the associations detected are mostly weak to moderate, suggesting that additional data, as well as more advanced models, will be required to model the increasingly complex relationships between external factors and infant growth in a more effective manner. In future work, it may be useful to apply more intricate multiomics modeling approaches^29^ to account for the different information densities within the different data modalities (for example, the microbiome is more sparse than other modalities). Finally, in this work, we focused solely on infant head circumference; however, the approach could equally be applied to other anthropometric outcomes such as infant weight, height, or growth trajectories. As these outcomes are tightly related, state-of-the-art multitask models^58^, as well as further concentrating on the interaction of mothers and their infants^59^ may be of particular interest.

## Conclusion

Using a systems biology approach to investigate multiple HM components simultaneously together with maternal, infant, and environmental data, we identified well-established pathways with potential practical implications (e.g., an association between maternal fish intake and HM DHA, which is connected to infant head circumference). These pathways, as well as our holistic machine learning based approach to understanding infant development in the context of head circumference, set the foundation for further analyses with additional dyads, HM analytes, and clinical outcomes. Within the CHILD Cohort Study and IMiC Consortium, this will include additional features such as the HM proteome, metabolome, and microbiome, as well as the gut microbiome and additional health outcomes, including linear growth, weight gain, asthma, and obesity. These additional modalities, outcomes, and increased sample sizes will enable the application of state-of-the-art multi-modal multi-task machine learning^58^ to jointly model, integrate, exploit, and understand relationships between the different HM components, other modalities and infant outcomes, paving the way for unprecedented insights into infant development.

## Materials and methods

### Study design and data

We used a systems biology approach to investigate multiple HM components together with maternal, infant, and environmental data from mother-infant dyads in the CHILD cohort. CHILD is an ongoing general population pregnancy cohort of 3624 families recruited in 2009–2012 across four Canadian centers (Vancouver, Edmonton, Manitoba, and Toronto)^35^. The study was approved by the Human Research Ethics Boards at McMaster University, University of Manitoba, University of Alberta, University of British Columbia, and SickKids Hospital, and carried out in accordance with relevant guidelines and regulations. All participants provided written informed consent at enrollment. Raw data and processed data will be available with appropriate permissions from the CHILD Cohort Study: https://childstudy.ca/for-researchers/data-access/.

HM samples were collected at 3–4 months postpartum during a home visit^36^. Briefly, mothers collected (hand expression preferred; pump expression accepted) and mixed foremilk and hindmilk from multiple feedings over a 24-h period and kept the sample refrigerated (for no more than 24 h) until it was collected by study staff and transported on ice to the laboratory for aliquoting and storage at – 80 °C until analysis. While some degradation of some components is possible within this 24-h period, current literature indicates that, aside from nucleic acids (which were not analyzed in our study), “refrigeration for up to 72 h keeps most constituents intact and limits lipolysis and bacterial growth”^60^. This sampling protocol could have increased the risk of (potentially selective) degradation of some HM components. However, the CHILD study opted to have mothers collect and refrigerate samples over a 24 h period in order to collect a “daily average” profile, which is important for HM components that are known to fluctuate diurnally. The HM subset (n = 1200) was originally selected to enrich for dyads with allergy and obesity phenotypes, plus healthy controls^38^. A subset of 1022 dyads with available HM data and head circumference measurements at both 3 months and 1 year was included in this analysis (see Supplementary Fig. S4 for a corresponding flow chart).

Head circumference was measured by trained and certified study staff at a 3 month home visit and at a 1 year clinical assessment. Head circumference was measured by taking a maximum of three repetitions. We used the z-score for age to represent head circumference in our study according to WHO standards^61^. The CHILD study excluded premature infants and those born with congenital anomalies, including macrocephaly and microcephaly. Genetic conditions associated with HC were not considered for exclusion.

HM data included: 3 hormones (leptin, insulin, and adiponectin) measured using enzyme-linked immunosorbent assay^39^, 19 HMOs measured using high-performance liquid chromatography^37,62^, 28 fatty acids measured using gas chromatography^38^, and 28 immunomodulators measured using immunoassays. Immunoglobulin levels and selected cytokines and chemokines were measured by Luminex multiplex assays: a panel of 24 analytes was analyzed using premixed multianalyte kits according to the manufacturers' recommendations and acquired by Luminex 200 (Bio-Rad), with calibration and standard controls. The Luminex kits used were R&D LXSAHM and Thermofisher EPX070-10818-901, EPX010-12283-901, and PPX-09-MX2W79V. Sandwich ELISA assays were used to assess total IgA (e-bioscience 88-50600-88), TGF-β1 (R&D Dy240), and TGF-β2 (R&D Dy241). We integrated these HM datasets with data reflecting maternal sociodemographic characteristics (e.g., age, marital status, education; n = 36 features), health (e.g., past and present chronic conditions; n = 84), diet (e.g., food and nutrient intakes and dietary patterns; n = 207)^63,64^, and body composition (e.g., height, weight, body mass index; n = 6); infant health (e.g., infections, colds, fevers, chronic conditions, medical visits; n = 87) and feeding practices (e.g., breastfeeding exclusivity and duration, introduction of formula milks and solid foods; n = 27); and the home environment (e.g., types of flooring, furniture and cleaning products; n = 31). For maternal BMI we refer to a best estimate of pre-pregnancy BMI. This estimate is based on height at 1 year postpartum (measured by study staff) and either self-reported pre-pregnancy weight or (if the mother could not recall) measured weight at 1 year postpartum. A complete list of features is provided in Supplemental Table S1. These features were used collectively to predict head circumference (z-score for age) measured by study staff at 3 months and 1 year using a flexible tape measure wrapped snugly around the widest possible circumference (average of three repeat measurements). The final number of features used to predict head circumference was 498 at 3 months and 582 at 1 year (some features like “the number of people in the house at 1 year” were not valid to use for predicting head circumference at 3 months).

### Predictive multi-omics modeling

We aimed to combine the previously mentioned data sources (HM oligosaccharides, fatty acids, hormones and immunomodulators; maternal demographic, health and dietary information, infant morbidities, feeding data, and home environment information) to collectively predict infant head circumference at 3 months and 1 year. To achieve this, we first align the different modalities across all infants and concatenate them into an integrated feature matrix. We then built models and evaluated them for both timepoints separately. For each timepoint, we employ a fivefold cross-validation scheme and collect predictions across all five folds to then calculate the significance of the prediction measured by Spearman’s rank correlation. The cross-validation scheme ensures that, even if overfitting on the training set occurs, the scores are reported on out of sample instances from the test set. In each fold, individually, we independently impute missing values using the median, and standard scale features. We kept all variables independent of their missing value count to preserve rare and seldomly recorded events like epilepsy or previous cancer therapy for the model to use. Imputation and scaling are derived from each fold’s training data and applied to the respective test data. We repeated this procedure 50 times and visualized the mean and standard deviation of the corresponding negative $lo{g}_{10}$ p-values in Fig. 1B. We also use the same set up to predict head circumference from individual data modalities to understand their contribution and association with head circumference. Note that milk immunomodulator and hormone modalities were excluded from model building since they were only available for less than one-third of the data. Additionally, we only include variables that have been measured at 3 months or before. For the models, we used linear and non-linear machine learning algorithms (Ridge regression and Support Vector Machines). The former has the potential to cope better with large amounts of features, even if features are highly correlated. The latter allows us to model more complex relationships between features and outcomes. For both approaches, the data matrix $X$ contains all features concatenated across all data modalities for all available mother-infant-dyads, while $y$ represents the head circumference either after three months or 1 year.

For ridge regression^65^, the goal is to derive coefficients $\beta$ for each feature in $X$ minimize the overall difference from $y$:

$$L(\beta )=|| y -X\beta |{{|}_{2}^{2} }.$$

However, this approach is not ideal for the analysis of the highly interrelated multi-modality data set, because it would select only representatives of communities of correlated features while disregarding highly correlated but potentially relevant features. To address this limitation, ${L}_{2}$ regularization is applied on $\beta$ to allow the inclusion of highly correlated measurements:

$${\beta }_{ridge}=argmi{n}_{\beta } || y -X\beta |{{|}_{2}^{2} +\lambda || \beta |{|}_{2}^{2}}$$

Here $\lambda$ specifies the regularization strength and is selected via nested cross-validation in each fold separately. That is, for each outer fold, the training set is split into inner folds via a leave-one-out procedure with a respective training and testing set each. For each inner fold we fit (inner training set) and test (inner test set/sample) ridge regression models with a set of parameters $\lambda \in \{\mathrm{0.1,1.0,10.0}\}$. The best parameter $\lambda$ according to mean performance (negative mean squared error) across the inner fold is selected to train the final model for the outer fold (the model is retrained on the complete outer training set).

In addition to ridge regression, we employ support vector regression^66^ with a radial basis function (RBF) kernel $K$ in order to capture more complex relationships between features and head circumference:

$$K({x}_{1},{x}_{2})=exp(-\frac{||{x}_{1}-{x}_{2}|{|}^{2}}{2{\sigma }^{2}})=exp(-\gamma ||{x}_{1}-{x}_{2}|{|}^{2})$$

Here, ${\sigma }^{2}$ represents the variance of the Gaussian distribution underlying the kernel, and $\gamma = \frac{1}{2{\sigma }^{2}}$. The use of this kernel allows the SVR model to project data points into an infinite dimensional space that enables learning nonlinear relationships between features in $X$ and outcome, where $\gamma$ specifies the radius of influence of each support vector/training sample on the final function. Here, $\gamma$ was set to $\frac{1}{{n}_{features}VAR(X)}$. In addition, SVMs allow to specify a parameter $C$ that allows adjusting how strongly outliers are taken into account. In our experiments $C$ is set to 1.

On a programmatic level, the Scikit-Learn (version 0.23.2) Python (version 3.7.8) package was used to train the models^67^.

### Univariate analysis and visualization

To understand univariate associations of individual features with head circumference, we calculated Kendall’s Tau for numeric features and Wilcoxon rank sum test for binary variables. The top associated features at 3 months are shown in Table 2, and the top features at 1 year are shown in Table 3. The corresponding p-values were corrected for false discovery rates using the Benjamini–Hochberg procedure. We furthermore give an overview of the association of head circumference to all features as correlation networks (cf. Figs. 2 and 3). To calculate 2-d coordinates for each variable, we first imputed all missing values using the respective medians. Then we calculated the correlation matrix between all features using Spearman correlation. Based on the absolute values of this correlation matrix, features are then placed in a 2-d plane using t-distributed stochastic neighbor embeddings (t-SNE)^68^. Thus, the closer to features, the more similar they are with regard to their correlation structure. We provide the same statistics for milk components, specifically in Tables 4 and 5, as well as Figs. 4 and 5.

### Supplementary Information

Supplementary Information.

## Data Availability

Raw data and processed data will be made available with appropriate permissions from the CHILD Cohort Study: https://childstudy.ca/for-researchers/data-access/. The source code is publicly available at http://nalab.stanford.edu/infant-growth-multiomics.
