# Supplementary material for: Multimodal machine learning for modeling infant head circumference, mothers’ milk composition, and their shared environment
Source: Sci Rep. 2024 Feb 5;14:2977. doi: 10.1038/s41598-024-52323-w (PMC10844250; doi:10.1038/s41598-024-52323-w)
Supplement: Supplementary file 1 — Supplementary Information. [file 41598_2024_52323_MOESM1_ESM.pdf]

Supplementary Material

Multimodal machine learning for modeling infant head circumference, mothers' milk composition, and their shared environment

Martin Becker[1,10], Kelsey Fehr[1,2,3,4], Stephanie Goguen[1,2,3,4], Kozeta Miliku[5,6], Catherine Field[7], Bianca Robertson[11], Chloe Yonemitsu[11], Lars Bode[1,11], Elinor Simons[4], Jean Marshall[8], Bassel Dawod[8], Piushkumar Mandhane[7], Stuart E. Turvey[9], Theo J. Moraes[12], Padmaja Subbarao[5,6,12], Natalie Rodriguez[1,2,3,4], Nima Aghaeepour[1,10,*], Meghan B. Azad[1,2,3,4,*]

[1] *International Milk Composition (IMiC) Consortium*

[2] *Manitoba Interdisciplinary Lactation Centre (MILC)*
[3] *Children's Hospital Research Institute of Manitoba*
[4] *University of Manitoba, Winnipeg, R3E3P4, Canada*

[5] *University of Toronto, Toronto, M5S 1A8, Canada*
[6] *McMaster University, Hamilton, M5S 1A8, Canada*
[7] *University of Alberta, Edmonton, T6G 2E1, Canada*
[8] *Dalhousie University, Halifax, B3H4R2, Canada*
[9] *UBC, Vancouver, V5Z4H4, Canada*

[10] *Stanford University, Stanford, 94305, USA*
[11] *University of California, San Diego, La Jolla, CA 92093, USA*

[12] *SickKids, Toronto, M5G 0A4, Canada*

Corresponding Authors:
Meghan Azad (meghan.azad@umanitoba.ca) - for CHILD cohort and IMiC consortium data
Nima Aghaeepour (naghaeep@stanford.edu) - for data analysis methods and code

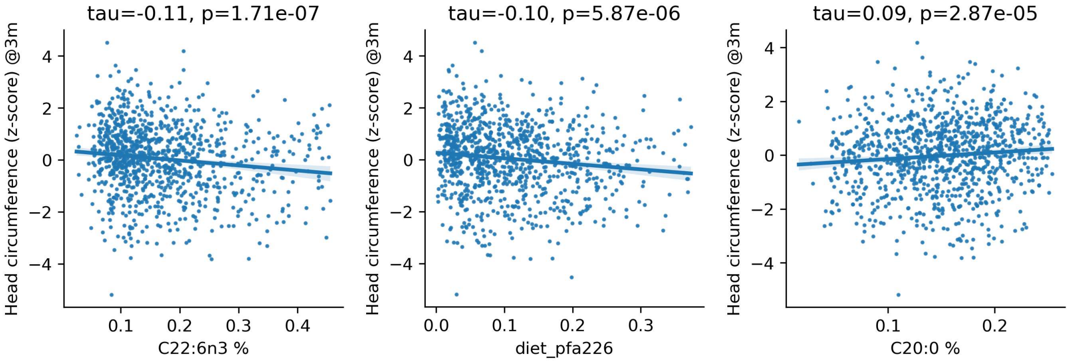

Supplementary Figure S1. Selected univariate relations to head circumference at 3 months. The plots show the feature on the x-axis and the head circumference at three months on the y-axis. Kendall's tau and p-value in the panel titles are calculated based on all available data points. Data points with values greater or lesser than three standard deviations were excluded for both axes. Regression lines are shown in each plot.

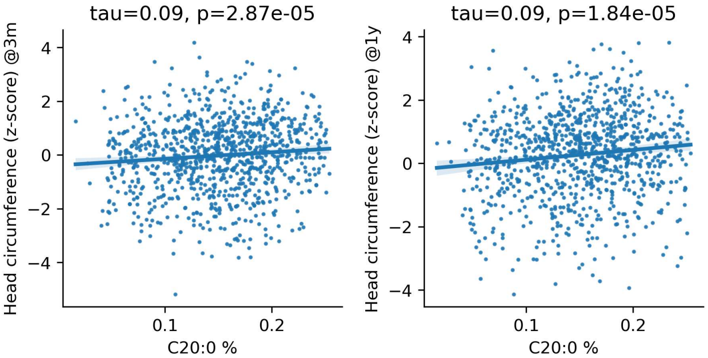

Supplementary Figure S2. Univariate relation of C20:0 % to head circumference at 3 months and 1 year. The plots show the feature on the x-axis and the head circumference at 3 months and 1 year on the y-axis, respectively. Kendall's tau and p-value in the panel titles are calculated based on all available data points. Data points with values greater or lesser than three standard deviations were excluded for both axes. Regression lines are shown in each plot.

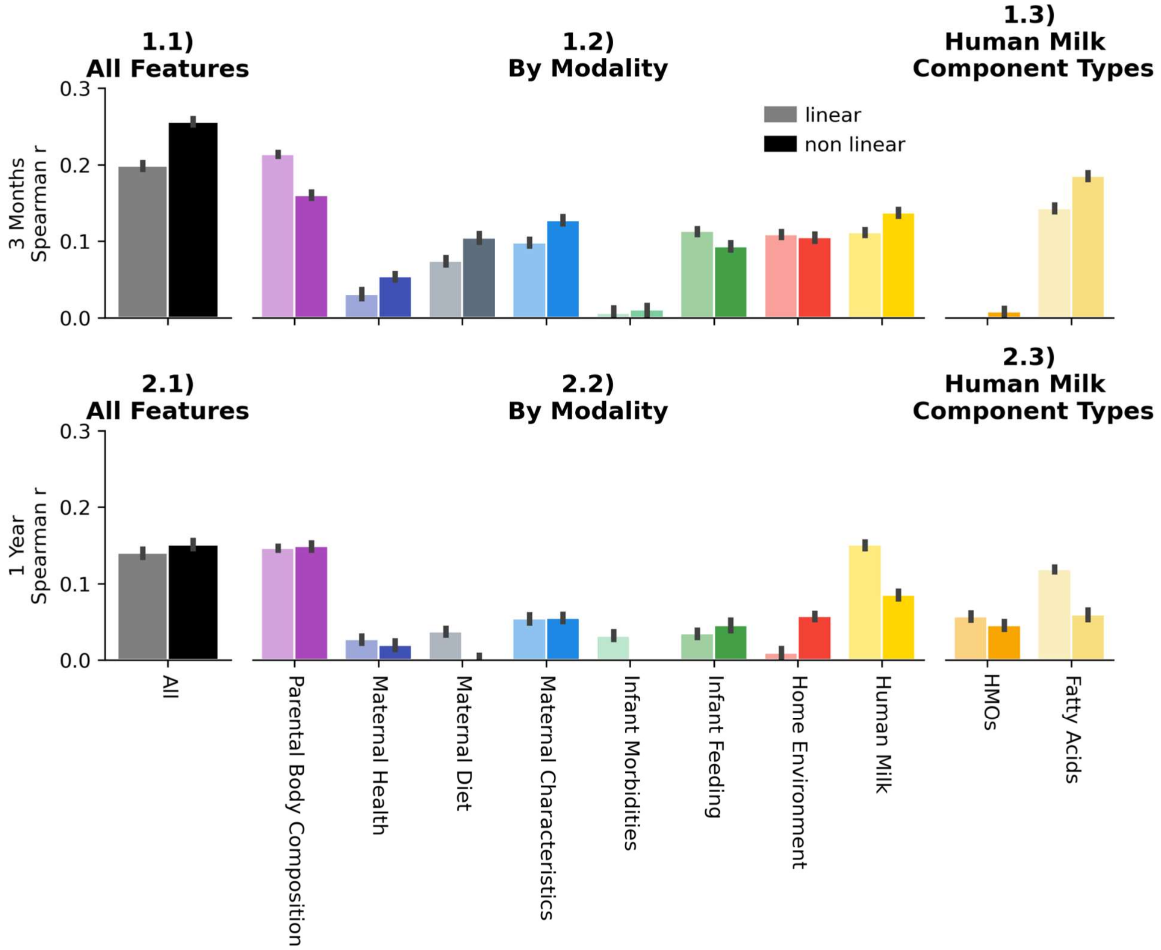

Supplemental Figure S3. Predictive modeling of head circumference at 3 months and 1 year using CHILD Cohort Study data (n=1014 mother-infant dyads; n=672 features): Spearman r values. Summary results from linear (ridge regression) and nonlinear (support vector machines) models using different combinations of features for prediction. Bars indicate the predictive power for each data subset and model measured by Spearman r between the predicted and actual head circumference. Spearman's r values indicate the strength of association based on the monotonicity of the relation between head circumference and the predicted head circumference. Results demonstrate that combining all features into a multi-model model increases predictive power, particularly at 3 months. Predicting head circumference further into the future (1 year) is more challenging than short-term predictions (3 months). Human milk components, particularly fatty acids, are predictive at 3 months.

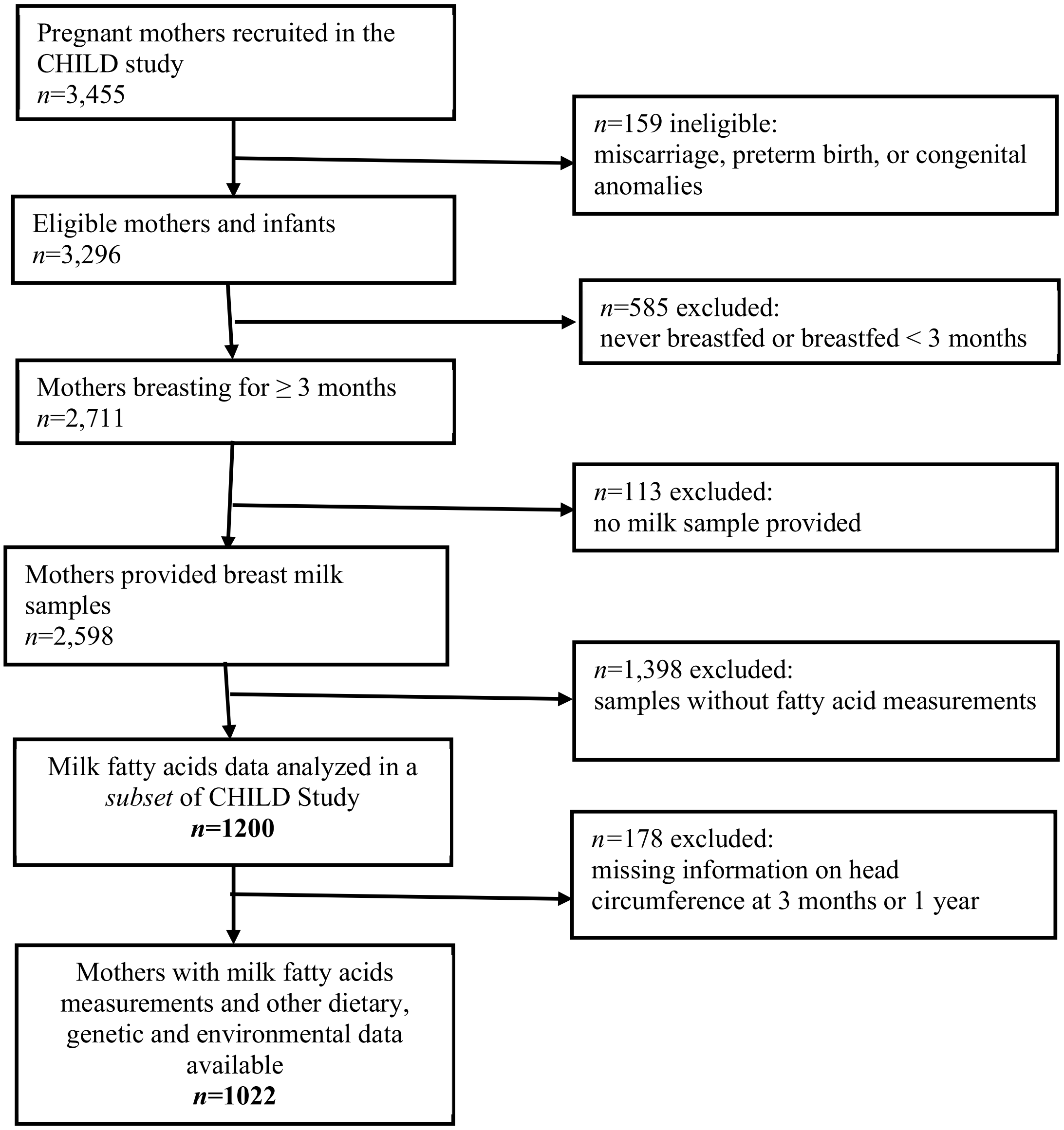

Supplementary Figure S4. Flowchart of the study participants

Supplemental Table S1. Variables used in prediction models for infant head circumference in the CHILD Cohort, sorted by modality and category. Short Names correspond to labels in Figures 3-6 and Tables 2-5.

Modality	Category	Short Name	Long Name
Home Environment	Number of people in house	adults_home_pg	Number of Adults living in home
Home Environment	Number of people in house	kids_home_pg	Number of Children living in home
Home Environment	Number of people in house	adults_home_1y	Number of Adults living in home
Home Environment	Number of people in house	kids_home_1y	Number of Children living in home
Home Environment	Number of bedrooms	rooms_pg	Rooms in home
Home Environment	Number of bedrooms	rooms_3m	3 month rooms in home
Home Environment	Heating & cooking	fuel_gas	Fuel in home - Gas/Propane
Home Environment	Heating & cooking	fuel_oil	Fuel in home - Oil
Home Environment	Heating & cooking	fuel_electric	Fuel in home - Electric
Home Environment	Heating & cooking	fuel_wood_pellet	Fuel in home - Wood/Pellet
Home Environment	Heating & cooking	fuel_dont know	Fuel in home - Don't know
Home Environment	Heating & cooking	fuel_other	Fuel in home - Other
Home Environment	Heating & cooking	fuel_other_spec	Fuel in home - Specify
Home Environment	Heating & cooking	wood_stove_fireplace	Prenatal wood buring stove or fireplace
Home Environment	Heating & cooking	stove	Prenatal type of stove
Home Environment	Heating & cooking	stove_other_spec	Prenatal type of stove, specify
Home Environment	Other smokers in house	smoke_home	Smoke home
Home Environment	Flooring	floor_installed_carpet	Floor type - Installed Carpet
Home Environment	Flooring	floor_hard_vinyl	Floor type - Hard vinyl
Home Environment	Flooring	floow_laminate	Floor type - Laminate
Home Environment	Flooring	floor_area_rug	Floor type - Area rug
Home Environment	Flooring	floor_soft_vinyl	Floor type - Soft vinyl
Home Environment	Flooring	floor_hardwood	Floor type - Hardwood
Home Environment	Flooring	floor_other	Floor type - Other
Home Environment	Flooring	floor_ceramic_tiles	Floor type - Ceramic tiles
Home Environment	Flooring	floor_other_spec	Floor type - Other specify
Home Environment	Flooring	age_floor	Age of floor
Home Environment	Animals (pets)	pet_pg	Prenatal pet owner
Home Environment	Animals (pets)	dog_pg	Prenatal dog owner
Home Environment	Animals (pets)	dog_num_pg	Prenatal number of dogs
Home Environment	Animals (pets)	cat_pg	Prenatal cat owner
Home Environment	Animals (pets)	cat_num_pg	Prenatal number of cats
Home Environment	Animals (pets)	furry_pets_pg	Prenatal other furry pets
Home Environment	Animals (pets)	pet_type_pg	Prenatal type of pet
Home Environment	Animals (pets)	num_pets_pg	Prenatal number of pets
Maternal Characteristics	Maternal smoking	smoke_often	Mother prenatal smoking
Maternal Characteristics	Maternal smoking	smoke_avg	Mother prenatal smoking per day
Maternal Characteristics	Maternal smoking	smoke_1y	Mother prental smoking 1 year
Maternal Characteristics	Maternal smoking	smoke_age	Mother prenatal smoking regularly age
Maternal Characteristics	Maternal smoking	smoke_prior_pg_start_stop	Mother prenatal smoking stop restart prior preg
Maternal Characteristics	Maternal smoking	smoke_prio_pg_stop	Mother prenatal stop smoking age prior preg
Maternal Characteristics	Maternal smoking	smoke_curr_pg_stop_avg	Mother prenatal stop smoking per day
Maternal Characteristics	Maternal smoking	smoke_curr_pg_stop	Mother prenatal stop smoking current preg
Maternal Characteristics	Maternal smoking	smoke_curr_pg_stop_wk	Mother prenatal stop smoking current preg weeks
Maternal Characteristics	Maternal smoking	smoke_curr_pg_avg	Mother prenatal smoke per day stopped
Maternal Characteristics	Maternal smoking	smoke_curr_pg_cut	Mother prenatal current preg smoking cut down
Maternal Characteristics	Maternal smoking	smoke_curr_pg_cut_wk	Mother prenatal current preg smoking weeks preg
Maternal Characteristics	Maternal smoking	smoke_curr_pg_cut_avg	Mother prentatl smoke average
Maternal Characteristics	Maternal smoking	smoke_often_pres	Mother prenatal smoke
Maternal Characteristics	Maternal smoking	smoke_per_day	Mother 1 year number of smokes
Maternal Characteristics	Maternal smoking	mom_smoke_prenatal	Mother smoking status during pregnancy
Maternal Characteristics	Maternal smoking	homesmokers	Do anyone smoke in the home
Maternal Characteristics	Age	DOB_mom	Date of birth for mother
Maternal Characteristics	Age	mom_age	Mother's age at birth of child

Maternal Characteristics	Age	age_mom	Mother's age at birth of child
Maternal Characteristics	Parity	older_sibs	Older siblings
Maternal Characteristics	Gravidity	gravida	Gravida
Maternal Characteristics	Season of birth	season_birth	Season of birth of child
Maternal Characteristics	Socioeconomic status	mom_edu	Maternal education level
Maternal Characteristics	Socioeconomic status	mom_edu_other	Other Maternal education level
Maternal Characteristics	Socioeconomic status	mom_edu_years	Maternal years of education
Maternal Characteristics	Socioeconomic status	data_edu	Father education level
Maternal Characteristics	Socioeconomic status	data_edu_year	Father years of education
Maternal Characteristics	Socioeconomic status	total_income	SES total income
Maternal Characteristics	Socioeconomic status	gov_source	SES government source
Maternal Characteristics	Socioeconomic status	SES_community	Perceived status in community
Maternal Characteristics	Socioeconomic status	SES_Canada	Perceived status in Canada
Maternal Characteristics	Socioeconomic status	home_owner	Homeowner
Maternal Characteristics	Socioeconomic status	mom_marital	Mother's martial status
Maternal Characteristics	Socioeconomic status	mom_marital_status	Mother's martial status
Maternal Characteristics	Delivery method	birthmode3	Mode of delivery
Maternal Characteristics	Delivery method	birthmode3_C-elective	birthmode3_C-elective
Maternal Characteristics	Delivery method	birthmode3_C-emergent	birthmode3_C-emergent
Maternal Characteristics	Delivery method	birthmode3_Vaginal	birthmode3_Vaginal
Maternal Characteristics	Delivery method	birthmode3_C-any	birthmode3_C-any
Maternal Body Composition	general	mom_bmi_best	Mother's BMI
Maternal Body Composition	general	mom_Height_cm	Mother's height
Maternal Body Composition	general	dad_Height_cm	Father's height
Maternal Body Composition	general	dad_Weight_kg	Fathers weight
Maternal Body Composition	Gestational weight gain	gest_wtgain	Gestation Weight Gain
Maternal Body Composition	Postpartum BMI	mom_BMI_1y	Mother's BMI at 1 y
Maternal Health	Gestational diabetes	GDM_CHILD	Gestational Diabetes
Maternal Health	Pre-eclampsia	pg_preeclampsia	Preeclampsia
Maternal Health	Other pregnancy complications	pg_bleeding	Bleeding
Maternal Health	Other pregnancy complications	pg_nausea	Nausea
Maternal Health	Other pregnancy complications	pg_infections	Infections
Maternal Health	Other pregnancy complications	pg_cardiac	Cardian disorders
Maternal Health	Other pregnancy complications	pg_hypertension	Hypertension
Maternal Health	Other pregnancy complications	pg_hypotension	Hypotention
Maternal Health	Other pregnancy complications	pg_notrecorded	Conditions not recorded
Maternal Health	Other pregnancy complications	pg_none	No conditions
Maternal Health	Other pregnancy complications	pg_highBP	High blood pressure during pregnancy
Maternal Health	Other pregnancy complications	pg_UTI	Urinary tract infection during pregnancy
Maternal Health	Other pregnancy complications	pg_sev_morn_sick	Severe morning sickness after 1st trimester during pregnancy
Maternal Health	Other pregnancy complications	pg_diarrhea	Diarrhea during pregnancy
Maternal Health	Other pregnancy complications	pg_high_choles	High Cholesterol during pregnancy
Maternal Health	Other pregnancy complications	pg_convulsions	Convulsions during pregnancy
Maternal Health	Other pregnancy complications	pg_diabetes	Diabetes blood sugar) during pregnancy

Maternal Health	Other pregnancy complications	pg_fever	Fever during pregnancy
Maternal Health	Other pregnancy complications	pg_cold_flu	old(s) or Flu during pregnancy
Maternal Health	Other pregnancy complications	pg_cold_sore	Cold sores during pregnancy
Maternal Health	Other pregnancy complications	pg_chest_inf_pneumonia	Chest infection/Pneumonia during pregnancy
Maternal Health	Other pregnancy complications	pg_STD	STD during pregnancy
Maternal Health	Other pregnancy complications	pg_yeast_infec	Yeast infection during pregnancy
Maternal Health	Other pregnancy complications	pg_ose	None ose conditions during pregnancy
Maternal Health	Other pregnancy complications	pg_other	Other conditions during pregnancy
Maternal Health	Other pregnancy complications	pg_other_spec	Othe spec fication during pregnancy
Maternal Health	Depression/Stress	psssumr_pre36wk	Mother's stress at 36 weeks pregnant
Maternal Health	Depression/Stress	csedsumr_pre36wk	Mother's depression at 36 weeks pregnant
Maternal Health	Depression/Stress	psssumr_pre18wk	Mother's stress at 18 weeks pregnant
Maternal Health	Depression/Stress	csedsumr_pre18wk	Mother's depression at 18 weeks
Maternal Health	Depression/Stress	psssumr_6m	Mother's stress at 6 months postpartum
Maternal Health	Depression/Stress	csedsumr_6m	Mother's drepression at 6 months postpartum
Maternal Health	Depression/Stress	psssumr_12m	Mother's stress at 1 year postpartum
Maternal Health	Depression/Stress	csedsumr_12m	Mother's depression at 1 year postpartum
Maternal Health	Depression/Stress	mom_dep_pg	Mother's prenatal depression
Maternal Health	Depression/Stress	mommed_dep_pg	Mother's prenatal depression Medication
Maternal Health	Depression/Stress	mom_dep_1y	Mother's depression at 1 year postpartum
Maternal Health	Depression/Stress	mommed_dep_1y	Mother's depression at 1 year postpartum medication
Maternal Health	Asthma	mom_asthma_ever	Mother ever had asthma
Maternal Health	Asthma	mom_asthma_md	Mother ever diagnosed with asthma
Maternal Health	Asthma	mom_asthmaRx_ever	Mother's receive treatment of asthma/wheezing
Maternal Health	Asthma	mom_asthmaRx_pg	Mother receive treatment of asthma/wheezing in the past 12 months
Maternal Health	Other health conditions	mom_atopy_ever	Mother ever had allergies
Maternal Health	Other health conditions	mom_atopy	Mother ever have atopy
Maternal Health	Other health conditions	mom_foodallergy	Mother ever had food allergy
Maternal Health	Other health conditions	mom_skinallergy	Mother ever had skin allergy
Maternal Health	Other health conditions	mom_anemia	Mother have anemia, prenatal
Maternal Health	Other health conditions	mommed_anemia	Mother medication for anemia, prenatal
Maternal Health	Other health conditions	mom_diabetes	Mother have diabetes, prenatal
Maternal Health	Other health conditions	mommed_diabetes	Mother medication for diabetes, prenatal
Maternal Health	Other health conditions	mom_highBP	Mother have high blood pressure
Maternal Health	Other health conditions	mommed_highBP	Mother medication for high blood pressure, prenatal
Maternal Health	Other health conditions	mom_IBS	Mother have IBS, prenatal
Maternal Health	Other health conditions	mommed_IBS	Mother medication for IBS, prenatal
Maternal Health	Other health conditions	mom_IBD	Mother have IBD, prenatal
Maternal Health	Other health conditions	mommed_IBD	Mother medication for IBD, prenatal
Maternal Health	Other health conditions	mom_highcholesterol	Mother have high cholestorl, prenatal
Maternal Health	Other health conditions	mommed_highcholesterol	Mother mediation for high cholestorl, prenatal
Maternal Health	Other health conditions	mom_reflux	Mother have heartburn/reflux, prenatal
Maternal Health	Other health conditions	mommed_reflux	Mother medication for heartburn/reflux, prenatal
Maternal Health	Other health conditions	mom_recurrentUTI	Mother have recurrent urinary infections, prenatal
Maternal Health	Other health conditions	mommed_recurrentUTI	Mother medication for recurrent urinary infections, prenatal
Maternal Health	Other health conditions	mom_kidneydisease	Mother have kidney disease, prenatal

Maternal Health	Other health conditions	mommed_kidneydisease	Mother medication for kidney disease, prenatal
Maternal Health	Other health conditions	mom_osteoperosis	Mother have osteoporosis, prenatal
Maternal Health	Other health conditions	mommed_osteoperosis	Mother medication for osteoporosis, prenatal
Maternal Health	Other health conditions	mom_arthritis	Mother have arthritis, prenatal
Maternal Health	Other health conditions	mommed_arthritis	Mother medication for arthritis, prenatal
Maternal Health	Other health conditions	mom_bloodclots	Mother have blood clots, prenatal
Maternal Health	Other health conditions	mommed_bloodclots	Mother medication for blood clots, prenatal
Maternal Health	Other health conditions	mom_migraine	Mother have migraine headaches, prenatal
Maternal Health	Other health conditions	mommed_migraine	Mother medication for migraine headaches, prenatal
Maternal Health	Other health conditions	mom_heartdisease	Mother have heart disease, prenatal
Maternal Health	Other health conditions	mommed_heartdisease	Mother medication for heart disease, prenatal
Maternal Health	Other health conditions	mom_depression	Mother have depression, prenatal
Maternal Health	Other health conditions	mommed_depression	Mother medication for depression, prenatal
Maternal Health	Other health conditions	mom_epilepsy	Mother have epilepsy, prenatal
Maternal Health	Other health conditions	mommed_epilepsy	Mother medication for epilepsy, prenatal
Maternal Health	Other health conditions	mom_stroke	Mother have stroke, prenatal
Maternal Health	Other health conditions	mommed_stroke	Mother medication for stroke, prenatal
Maternal Health	Other health conditions	mom_cancer	Mother have cancer, prenatal
Maternal Health	Other health conditions	mommed_cancer_chemo	Mother have chemotherapy for cancer, prenatal
Maternal Health	Other health conditions	mommed_cancer_radiation	Mother have radiation therapy for cancer, prenatal
Maternal Health	Other health conditions	momother_health	Mother other health issues, prenatal
Maternal Health	Other health conditions	momother_med	Mother other health issues specify, prenatal
Maternal Health	Other health conditions	mommed	Mother other health issues medications, prenatal
Maternal Diet	General dietary assessment	ADD_SUGAR	Teaspoon equivalents of added sugars
Maternal Diet	General dietary assessment	diet_A_BEV	Total drinks of alcohol
Maternal Diet	General dietary assessment	diet_A_CAL	Calories from alcoholic beverages
Maternal Diet	General dietary assessment	diet_DISCFAT_OIL	Grams of discretionary Oil
Maternal Diet	General dietary assessment	diet_DISCFAT_SOL	Grams of discretionary Solid fat
Maternal Diet	General dietary assessment	diet_D_CHEESE	Number of cheese cup equivalents
Maternal Diet	General dietary assessment	diet_D_MILK	Number of milk cup equivalents
Maternal Diet	General dietary assessment	diet_D_TOTAL	Total number of milk group (milk, yogurt & cheese) cup equivalents
Maternal Diet	General dietary assessment	diet_D_TOT_SOYM	Total number of milk group (milk, yogurt & cheese) cup equivalents PLUS soy milk
Maternal Diet	General dietary assessment	diet_D_YOGURT	Number of yogurt cup equivalents
Maternal Diet	General dietary assessment	diet_F_CITMLB	Number of citrus, melon, berry cup equivalents
Maternal Diet	General dietary assessment	diet_F_NJ_CITMLB	Number of non-juice citrus, melon, berry cup equivalents
Maternal Diet	General dietary assessment	diet_F_NJ_OTHER	Number of other non-juice fruit cup equivalents
Maternal Diet	General dietary assessment	diet_F_NJ_TOTAL	Total number of non-juice fruit cup equivalents
Maternal Diet	General dietary assessment	diet_F_OTHER	Number of other fruit cup equivalents
Maternal Diet	General dietary assessment	diet_F_TOTAL	Total number of fruit cup equivalents
Maternal Diet	General dietary assessment	diet_G_NWHL	Number of non-whole grain ounce equivalents
Maternal Diet	General dietary assessment	diet_G_TOTAL	Total number of grain ounce equivalents
Maternal Diet	General dietary assessment	diet_G_WHL	Number of whole grain ounce equivalents
Maternal Diet	General dietary assessment	diet_LEGUMES	Number of cooked dry beans and peas cup equivalents
Maternal Diet	General dietary assessment	diet_M_EGG	Oz equivalents of lean meat from eggs
Maternal Diet	General dietary assessment	diet_M_FISH_HI	Oz cooked lean meat from fish, other seafood high in Omega-3
Maternal Diet	General dietary assessment	diet_M_FISH_LO	Oz cooked lean meat from fish, other seafood low in Omega-3
Maternal Diet	General dietary assessment	diet_M_FRANK	Oz cooked lean meat from franks, sausages, luncheon meats

Maternal Diet	General dietary assessment	diet_M_MEAT	Oz cooked lean meat from beef, pork, veal, lamb, and game
Maternal Diet	General dietary assessment	diet_M_MPF	Oz cooked lean meat from meat, poultry, fish
Maternal Diet	General dietary assessment	diet_M_NUTSD	Oz equivalents of lean meat from nuts and seeds
Maternal Diet	General dietary assessment	diet_M_ORGAN	Oz cooked lean meat from organ meats
Maternal Diet	General dietary assessment	diet_M_POULT	Oz cooked lean meat from chicken, poultry, and other poultry
Maternal Diet	General dietary assessment	diet_M_SOY	Oz equivalents of lean meat from soy product
Maternal Diet	General dietary assessment	diet_V_DRKGR	Number of dark-green vegetable cup equivalents
Maternal Diet	General dietary assessment	diet_V_ORANGE	Number of orange vegetable cup equivalents
Maternal Diet	General dietary assessment	diet_V_OTHER	Number of other vegetable cup equivalents
Maternal Diet	General dietary assessment	diet_V_POTATO	Number of white potato cup equivalents
Maternal Diet	General dietary assessment	diet_V_STARCY	Number of other starchy vegetable cup equivalents
Maternal Diet	General dietary assessment	diet_V_TOMATO	Number of tomato cup equivalents
Maternal Diet	General dietary assessment	diet_V_TOTAL	Total number of vegetable cup equivalents, excl legumes
Maternal Diet	General dietary assessment	diet_acesupot	Acesulfame Potassium (mg)
Maternal Diet	General dietary assessment	diet_addsugar	Added Sugars (by Available Carbohydrate) (g)
Maternal Diet	General dietary assessment	diet_alanine	Alanine (g)
Maternal Diet	General dietary assessment	diet_alcohol	Alcohol (g)
Maternal Diet	General dietary assessment	diet_alphacar	Alpha-Carotene (provitamin A carotenoid) (mcg)
Maternal Diet	General dietary assessment	diet_alphtoce	Total Vitamin E Activity (total alpha-tocopherol equivalents) (mg)
Maternal Diet	General dietary assessment	diet_alphtoco	Alpha-Tocopherol (mg)
Maternal Diet	General dietary assessment	diet_arginine	Arginine (g)
Maternal Diet	General dietary assessment	diet_ash	Ash (g)
Maternal Diet	General dietary assessment	diet_aspartam	Aspartame (mg)
Maternal Diet	General dietary assessment	diet_aspartic	Aspartic Acid (g)
Maternal Diet	General dietary assessment	diet_avcarb	Available Carbohydrate (g)
Maternal Diet	General dietary assessment	diet_betacar	Beta-Carotene (provitamin A carotenoid) (mcg)
Maternal Diet	General dietary assessment	diet_betacryp	Beta-Cryptoxanthin (provitamin A carotenoid) (mcg)
Maternal Diet	General dietary assessment	diet_betaine	Betaine (mg)
Maternal Diet	General dietary assessment	diet_betatoco	Beta-Tocopherol (mg)
Maternal Diet	General dietary assessment	diet_biochana	Biochanin A (mg)
Maternal Diet	General dietary assessment	diet_caffeine	Caffeine (mg)
Maternal Diet	General dietary assessment	diet_calcium	Calcium (mg)
Maternal Diet	General dietary assessment	diet_calories	Energy (kcal)
Maternal Diet	General dietary assessment	diet_carbo	Total Carbohydrate (g)
Maternal Diet	General dietary assessment	diet_cholest	Cholesterol (mg)
Maternal Diet	General dietary assessment	diet_choline	Choline (mg)
Maternal Diet	General dietary assessment	diet_clac9t11	CLA cis-9, trans-11 (g)
Maternal Diet	General dietary assessment	diet_clat10c12	CLA trans-10, cis-12 (g)
Maternal Diet	General dietary assessment	diet_copper	Copper (mg)
Maternal Diet	General dietary assessment	diet_coumest	Coumestrol (mg)
Maternal Diet	General dietary assessment	diet_cystine	Cystine (g)
Maternal Diet	General dietary assessment	diet_daidzein	Daidzein (mg)
Maternal Diet	General dietary assessment	diet_delttoco	Delta-Tocopherol (mg)
Maternal Diet	General dietary assessment	diet_erythr	Erythritol (g)
Maternal Diet	General dietary assessment	diet_fat	Total Fat (g)
Maternal Diet	General dietary assessment	diet_fiber	Total Dietary Fiber (g)
Maternal Diet	General dietary assessment	diet_fibh2o	Soluble Dietary Fiber (g)
Maternal Diet	General dietary assessment	diet_fibinso	Insoluble Dietary Fiber (g)
Maternal Diet	General dietary assessment	diet_fol_deqv	Dietary Folate Equivalents (mcg)
Maternal Diet	General dietary assessment	diet_fol_nat	Natural Folate (food folate) (mcg)
Maternal Diet	General dietary assessment	diet_fol_syn	Synthetic Folate (folic acid) (mcg)
Maternal Diet	General dietary assessment	diet_formontn	Formononetin (mg)
Maternal Diet	General dietary assessment	diet_fructose	Fructose (g)

Maternal Diet	General dietary assessment	diet_galactos	Galactose (g)
Maternal Diet	General dietary assessment	diet_gammtoco	Gamma-Tocopherol (mg)
Maternal Diet	General dietary assessment	diet_genistn	Genistein (mg)
Maternal Diet	General dietary assessment	diet_GLAC	Glycemic load based on available carb
Maternal Diet	General dietary assessment	diet_GLTC	Glycemic load based on total carb
Maternal Diet	General dietary assessment	diet_glucose	Glucose (g)
Maternal Diet	General dietary assessment	diet_glutamic	Glutamic Acid (g)
Maternal Diet	General dietary assessment	diet_glycine	Glycine (g)
Maternal Diet	General dietary assessment	diet_glycitn	Glycitein (mg)
Maternal Diet	General dietary assessment	diet_grams	Total Grams
Maternal Diet	General dietary assessment	diet_histidin	Histidine (g)
Maternal Diet	General dietary assessment	diet_inositol	Inositol (g)
Maternal Diet	General dietary assessment	diet_iron	Iron (mg)
Maternal Diet	General dietary assessment	diet_isoleuc	Isoleucine (g)
Maternal Diet	General dietary assessment	diet_isomalt	Isomalt (g)
Maternal Diet	General dietary assessment	diet_joules	Energy (kj)
Maternal Diet	General dietary assessment	diet_lactitol	Lactitol (g)
Maternal Diet	General dietary assessment	diet_lactose	Lactose (g)
Maternal Diet	General dietary assessment	diet_leucine	Leucine (g)
Maternal Diet	General dietary assessment	diet_lutzeax	Lutein + Zeaxanthin (mcg)
Maternal Diet	General dietary assessment	diet_lycopene	Lycopene (mcg)
Maternal Diet	General dietary assessment	diet_lysine	Lysine (g)
Maternal Diet	General dietary assessment	diet_magnes	Magnesium (mg)
Maternal Diet	General dietary assessment	diet_maltitol	Maltitol (g)
Maternal Diet	General dietary assessment	diet_maltose	Maltose (g)
Maternal Diet	General dietary assessment	diet_mangan	Manganese (mg)
Maternal Diet	General dietary assessment	diet_mannitol	Mannitol (g)
Maternal Diet	General dietary assessment	diet_methhis3	3-Methylhistidine (mg)
Maternal Diet	General dietary assessment	diet_methion	Methionine (g)
Maternal Diet	General dietary assessment	diet_mfa141	MUFA 14:1 (myristoleic acid) (g)
Maternal Diet	General dietary assessment	diet_mfa161	MUFA 16:1 (palmitoleic acid) (g)
Maternal Diet	General dietary assessment	diet_mfa181	MUFA 18:1 (oleic acid) (g)
Maternal Diet	General dietary assessment	diet_mfa201	MUFA 20:1 (gadoleic acid) (g)
Maternal Diet	General dietary assessment	diet_mfa221	MUFA 22:1 (erucic acid) (g)
Maternal Diet	General dietary assessment	diet_mfatot	Total Monounsaturated Fatty Acids (MUFA) (g)
Maternal Diet	General dietary assessment	diet_natoco	Natural Alpha-Tocopherol (RRR-alpha-tocopherol or d-alpha-tocopherol) (mg)
Maternal Diet	General dietary assessment	diet_nccglbr	NCC Glycemic Load (bread reference)
Maternal Diet	General dietary assessment	diet_nccglgr	NCC Glycemic Load (glucose reference)
Maternal Diet	General dietary assessment	diet_niacin	Niacin (vitamin B3) (mg)
Maternal Diet	General dietary assessment	diet_niacineq	Niacin Equivalents (mg)
Maternal Diet	General dietary assessment	diet_nitrogen	Nitrogen (g)
Maternal Diet	General dietary assessment	diet_omega3	Omega-3 Fatty Acids (g)
Maternal Diet	General dietary assessment	diet_oxalic	Oxalic Acid (mg)
Maternal Diet	General dietary assessment	diet_pantothe	Pantothenic acid (mg)
Maternal Diet	General dietary assessment	diet_pectins	Pectins (g)
Maternal Diet	General dietary assessment	diet_pfa182	PUFA 18:2 (linoleic acid) (g)
Maternal Diet	General dietary assessment	diet_pfa183	PUFA 18:3 (linolenic acid) (g)
Maternal Diet	General dietary assessment	diet_pfa184	PUFA 18:4 (parinaric acid) (g)
Maternal Diet	General dietary assessment	diet_pfa204	PUFA 20:4 (arachidonic acid) (g)
Maternal Diet	General dietary assessment	diet_pfa205	PUFA 20:5 (eicosapentaenoic acid [EPA]) (g)
Maternal Diet	General dietary assessment	diet_pfa225	PUFA 22:5 (docosapentaenoic acid [DPA]) (g)
Maternal Diet	General dietary assessment	diet_pfa226	PUFA 22:6 (docosahexaenoic acid [DHA]) (g)
Maternal Diet	General dietary assessment	diet_pfatot	Total Polyunsaturated Fatty Acids (PUFA) (g)
Maternal Diet	General dietary assessment	diet_phenylal	Phenylalanine (g)
Maternal Diet	General dietary assessment	diet_phosphor	Phosphorus (mg)
Maternal Diet	General dietary assessment	diet_phytic	Phytic Acid (mg)
Maternal Diet	General dietary assessment	diet_pinitol	Pinitol (g)
Maternal Diet	General dietary assessment	diet_potass	Potassium (mg)

Maternal Diet	General dietary assessment	diet_proline	Proline (g)
Maternal Diet	General dietary assessment	diet_protanim	Animal Protein (g)
Maternal Diet	General dietary assessment	diet_protein	Total Protein (g)
Maternal Diet	General dietary assessment	diet_protveg	Vegetable Protein (g)
Maternal Diet	General dietary assessment	diet_retinol	Retinol (mcg)
Maternal Diet	General dietary assessment	diet_ribofla	Riboflavin (vitamin B2) (mg)
Maternal Diet	General dietary assessment	diet_sacchar	Saccharin (mg)
Maternal Diet	General dietary assessment	diet_satoco	Synthetic Alpha-Tocopherol (all rac-alpha-tocopherol or dl-alpha-tocopherol) (mg)
Maternal Diet	General dietary assessment	diet_selenium	Selenium (mcg)
Maternal Diet	General dietary assessment	diet_serine	Serine (g)
Maternal Diet	General dietary assessment	diet_sfa100	SFA 10:0 (capric acid) (g)
Maternal Diet	General dietary assessment	diet_sfa120	SFA 12:0 (lauric acid) (g)
Maternal Diet	General dietary assessment	diet_sfa140	SFA 14:0 (myristic acid) (g)
Maternal Diet	General dietary assessment	diet_sfa160	SFA 16:0 (palmitic acid) (g)
Maternal Diet	General dietary assessment	diet_sfa170	SFA 17:0 (margaric acid) (g)
Maternal Diet	General dietary assessment	diet_sfa180	SFA 18:0 (stearic acid) (g)
Maternal Diet	General dietary assessment	diet_sfa200	SFA 20:0 (arachidic acid) (g)
Maternal Diet	General dietary assessment	diet_sfa220	SFA 22:0 (behenic acid) (g)
Maternal Diet	General dietary assessment	diet_sfa40	SFA 4:0 (butyric acid) (g)
Maternal Diet	General dietary assessment	diet_sfa60	SFA 6:0 (caproic acid) (g)
Maternal Diet	General dietary assessment	diet_sfa80	SFA 8:0 (caprylic acid) (g)
Maternal Diet	General dietary assessment	diet_sfatot	Total Saturated Fatty Acids (SFA) (g)
Maternal Diet	General dietary assessment	diet_sodium	Sodium (mg)
Maternal Diet	General dietary assessment	diet_sorbitol	Sorbitol (g)
Maternal Diet	General dietary assessment	diet_starch	Starch (g)
Maternal Diet	General dietary assessment	diet_sucpoly	Sucrose polyester (g)
Maternal Diet	General dietary assessment	diet_sucrlose	Sucralose (mg)
Maternal Diet	General dietary assessment	diet_sucrose	Sucrose (g)
Maternal Diet	General dietary assessment	diet_tagatose	Tagatose (mg)
Maternal Diet	General dietary assessment	diet_tfa161t	TRANS 16:1 (trans-hexadecenoic acid) (g)
Maternal Diet	General dietary assessment	diet_tfa181t	TRANS 18:1 (trans-octadecenoic acid [elaidic acid]) (g)
Maternal Diet	General dietary assessment	diet_tfa182t	TRANS 18:2 (trans-octadecadienoic acid [linolelaidic acid]; incl. c-t, t-c, t-t) (g)
Maternal Diet	General dietary assessment	diet_thiamin	Thiamin (vitamin B1) (mg)
Maternal Diet	General dietary assessment	diet_threonin	Threonine (g)
Maternal Diet	General dietary assessment	diet_totaltfa	Total Trans-Fatty Acids (TRANS) (g)
Maternal Diet	General dietary assessment	diet_totcla	Total Conjugated Linoleic Acid (CLA 18:2) (g)
Maternal Diet	General dietary assessment	diet_totfolat	Total Folate (mcg)
Maternal Diet	General dietary assessment	diet_totsugar	Total Sugars (g)
Maternal Diet	General dietary assessment	diet_tryptoph	Tryptophan (g)
Maternal Diet	General dietary assessment	diet_tyrosine	Tyrosine (g)
Maternal Diet	General dietary assessment	diet_valine	Valine (g)
Maternal Diet	General dietary assessment	diet_vita_iu	Total Vitamin A Activity (International Units) (IU)
Maternal Diet	General dietary assessment	diet_vita_rae	Total Vitamin A Activity (Retinol Activity Equivalents) (mcg)
Maternal Diet	General dietary assessment	diet_vita_re	Total Vitamin A Activity (Retinol Equivalents) (mcg)
Maternal Diet	General dietary assessment	diet_vitb12	Vitamin B-12 (cobalamin) (mcg)
Maternal Diet	General dietary assessment	diet_vitb6	Vitamin B-6 (pyridoxine, pyridoxyl, & pyridoxamine) (mg)
Maternal Diet	General dietary assessment	diet_vitc	Vitamin C (ascorbic acid) (mg)
Maternal Diet	General dietary assessment	diet_vitd	Vitamin D (calciferol) (mcg)
Maternal Diet	General dietary assessment	diet_vitd2	Vitamin D2 (ergocalciferol) (mcg)
Maternal Diet	General dietary assessment	diet_vitd3	Vitamin D3 (cholecalciferol) (mcg)
Maternal Diet	General dietary assessment	diet_vite_iu	Vitamin E (International Units) (IU)
Maternal Diet	General dietary assessment	diet_vitk	Vitamin K (phylloquinone) (mcg)
Maternal Diet	General dietary assessment	diet_water	Water (g)
Maternal Diet	General dietary assessment	diet_xylitol	Xylitol (g)
Maternal Diet	General dietary assessment	diet_zinc	Zinc (mg)
Maternal Diet	General dietary assessment	diet_frt5day	Daily Fruit Consump (5-A-Day Method)

Maternal Diet	General dietary assessment	diet_frtsumm	Daily Fruit Consump (Summation Method)
Maternal Diet	General dietary assessment	diet_veg5day	Daily Vegetable Consump (5-A-Day Method)
Maternal Diet	General dietary assessment	diet_vegsumm	Daily Vegetable Consump (Summation Meth)
Maternal Diet	General dietary assessment	hei1_total_fruit	Healthy Eating Index: Total fruit
Maternal Diet	General dietary assessment	hei2_whole_fruit	Healthy Eating Index: Whole fruit
Maternal Diet	General dietary assessment	hei3_total_veg	Healthy Eating Index: Total vegetables
Maternal Diet	General dietary assessment	hei4_greens_beans	Healthy Eating Index: TotalGreens and beans
Maternal Diet	General dietary assessment	hei5_whole_grain	Healthy Eating Index: Whole grains
Maternal Diet	General dietary assessment	hei6_dairy	Healthy Eating Index: Dairy
Maternal Diet	General dietary assessment	hei7_total_protien	Healthy Eating Index: Total protien foods
Maternal Diet	General dietary assessment	hei8_seafood_plat_protien	Healthy Eating Index: Seafood and plant proteins
Maternal Diet	General dietary assessment	hei9_FA	Healthy Eating Index: Fatty acids
Maternal Diet	General dietary assessment	hei10_grain	Healthy Eating Index: Refined grains
Maternal Diet	General dietary assessment	hei11_sodium	Healthy Eating Index: Sodium
Maternal Diet	General dietary assessment	hei12_empty_cal	Healthy Eating Index: Empty calories
Maternal Diet	General dietary assessment	hei2010	Total score of 12 Health Eating Index Components
Infant Feeding	Various	BFpump_3m	Breast milk from pump at 3 months postpartum
Infant Feeding	Various	BF_1m	Breastfeeding at 1 month
Infant Feeding	Various	BF_3m	Breastfeeding at 3 months
Infant Feeding	Various	BF_6m	Breastfeeding at 6 months
Infant Feeding	Various	BF_9m	Breastfeeding at 9 months
Infant Feeding	Various	BF_12m	Breastfeeding at 12 months
Infant Feeding	Various	BF_18m	Breastfeeding at 18 months
Infant Feeding	Various	BF_24m	Breasfeeding at 24 months
Infant Feeding	Various	BF_hosp_any	Breastfeeding in hospital (from questionniare and hospital charts)
Infant Feeding	Various	FF_hosp_any	Fromula in hospital (from questionnaire and hospital chart review)
Infant Feeding	Various	FF_3m	Forumla fed before 3 months
Infant Feeding	Various	FF_4m	Forumla fed before 4 months
Infant Feeding	Various	FF_6m	Forumla fed before 6 months
Infant Feeding	Various	BF_ever	Ever breastfed (after hosptial)
Infant Feeding	Various	LBF_ever	Lifetime ever breastfed
Infant Feeding	Various	EBF_hosp	Exclusive Breastfeeding in Hospital
Infant Feeding	Various	EBF_3m	Exclusive Breastfeeding at 3 months
Infant Feeding	Various	EBF_6m	Exclusive Breastfeeding at 6 months
Infant Feeding	Various	EBF_duration	Duration of Exclusive Breastfeeding (Months)
Infant Feeding	Various	LEBF_3m	Lifetime Exclusive Breastfeeding at 3 months
Infant Feeding	Various	LEBF_6m	Lifetime Exclusive Breastfeeding at 6 months
Infant Feeding	Various	LEBF_duration	Duration of Lifetime Exclusive Breastfeeding (Months)
Infant Feeding	Various	BF_3m_status	Breastfeeding status at 3 months
Infant Feeding	Various	BF_6m_status	Breastfeeding status at 6 months
Infant Feeding	Various	LBF_3m_status	Lifetime Breastfeeding status at 3 months
Infant Feeding	Various	LBF_6m_status	Lifetime Breastfeeding status at 6 months
Infant Feeding	Various	BF_duration_imp_12m	BF_duration_imp_12m
Infant Feeding	Various	first_formula	First Introduction of Formula (months)
Infant Feeding	Various	first_solid	First Introduction of Solids (months
Infant Feeding	Various	diet_3m	Infant Diet at 3m - BFS: Breast, Formula, Solids (CAPITAL=YES, small=no)
Infant Feeding	Various	diet_6m	Infant Diet at 6m - BFS: Breast, Formula, Solids (CAPITAL=YES, small=no)
Infant Feeding	Various	solids_3m	Solids before 3m (yes=1)
Infant Feeding	Various	solids_4m	Solids before 4m
Infant Feeding	Various	solids_6m	Solids before 6m
Infant Feeding	Various	Lfirst_formula	Lifetime First Introduction of Formula
Infant Feeding	Various	BM_mode_3m	Mode of B(reast) M(ilk) feeding AT 3m, I(ndirect) or D(irect)

Infant Feeding	Various	Feed_mode4_3m	Direct/Indirect BreastFeeding, Formula Feeding at 3m
Infant Morbidities	Fever	fever_3m	Child have fever since birth
Infant Morbidities	Fever	worst_fever_3m	Worst fever last, birth
Infant Morbidities	Fever	temp_fever_3m	Highest temperature of fever, birth
Infant Morbidities	Fever	hightemp_fever_3m	Highest recorded temperature of fever,birth
Infant Morbidities	Fever	fever_cause_3m	Fever thought to be from, birth
Infant Morbidities	Fever	fever_6m	Child have fever between 3 and 6 months
Infant Morbidities	Fever	worst_fever_6m	Worst fever last, 3 and 6 months
Infant Morbidities	Fever	temp_fever_6m	Highest temperature of fever, 3 and 6 months
Infant Morbidities	Fever	hightemp_fever_6m	Highest recorded temperature of fever, 3 and 6 months
Infant Morbidities	Fever	fever_cause_6m	Fever thought to be from, 3 and 6 months
Infant Morbidities	Fever	fever_1y	Child have fever last 6 months
Infant Morbidities	Fever	worst_fever_1y	Worst fever last, last 6 months
Infant Morbidities	Fever	temp_fever_1y	Highest temperature of fever, last 6 months
Infant Morbidities	Fever	hightemp_fever_1y	Highest recorded temperature of fever, last 6 months
Infant Morbidities	Fever	fever_cause_1y	Fever thought to be from, last 6 months
Infant Morbidities	Diarrhea	cold1_diarreah_3m	Child cold 1 symptom, diarrhea at 3m
Infant Morbidities	Diarrhea	cold2_diarreah_3m	Child cold 2 symptom, diarrhea at 3m
Infant Morbidities	Diarrhea	cold1_diarreah_6m	Child cold 1 symptom, diarrhea at 6m
Infant Morbidities	Diarrhea	cold2_diarreah_6m	Child cold 2 symptom, diarrhea at 6m
Infant Morbidities	Diarrhea	cold1_diarreah_1y	Child cold 1 symptom, diarrhea at 1y
Infant Morbidities	Diarrhea	cold2_diarreah_1y	Child cold 2 symptom, diarrhea at 1y
Infant Morbidities	Hospital visits	hosp_3m	Child admitted to hosptial/stay since birth
Infant Morbidities	Hospital visits	hospadmin_3m	How many hosptial admissions/stays since birth
Infant Morbidities	Hospital visits	hospadmin_cold_3m	Child hospital stay, since birth - Bad cold
Infant Morbidities	Hospital visits	hospadmin_fever_3m	Child hospital stay, since birth - Fever
Infant Morbidities	Hospital visits	hospadmin_rash_3m	Child hospital stay, since birth - Rash
Infant Morbidities	Hospital visits	hospadmin_wz_3m	Child hospital stay, since birth - Wheezing episode
Infant Morbidities	Hospital visits	hospadmin_ear_3m	Child hospital stay - Ear infection, since birth
Infant Morbidities	Hospital visits	hospadmin_allergy_3m	Child hospital stay, since birth - Allergy
Infant Morbidities	Hospital visits	hospadmin_asthma_3m	Child hospital stay, since birth - Asthma
Infant Morbidities	Hospital visits	hospadmin_chest_3m	Child hospital stay, since birth - Chest
Infant Morbidities	Hospital visits	hospadmin_acci_3m	Child hospital stay, since birth - Accident
Infant Morbidities	Hospital visits	hospadmin_cough_3m	Child hospital stay, since birth - Coughing
Infant Morbidities	Hospital visits	hospadmin_other_3m	Child hospital stay, since birth - Other
Infant Morbidities	Hospital visits	hospstay_long_3m	Child's longest hopsital stay in days
Infant Morbidities	Hospital visits	hospstay_intubation_3m	Child hospital procedure, since birth - Intubation
Infant Morbidities	Hospital visits	hospstay_bloodtests_3m	Child hospital procedure, since birth - Blood tests
Infant Morbidities	Hospital visits	hospstay_facepuffer_3m	Child hospital procedure, since birth - Facemask or puffer
Infant Morbidities	Hospital visits	hospstay_IV_3m	Child hospital procedure, since birth - IV
Infant Morbidities	Hospital visits	hospstay_oxygen_3m	Child hospital procedure, since birth - Oxygen
Infant Morbidities	Hospital visits	hospstay_other_3m	Child hospital procedure, since birth - Other
Infant Morbidities	Hospital visits	hospstay_otherspec_3m	Child hospital procedure, since birth - Other, specify
Infant Morbidities	Hospital visits	hosp_ICU_3m	Child in ICU at any stay, since birth
Infant Morbidities	Hospital visits	hosp_coldcoughwz_3m	Child admitted to hosptial/stay since birth
Infant Morbidities	Hospital visits	hosp_coldcoughwz_numstay_3m	How many hosptial admissions/stays since birth
Infant Morbidities	Hospital visits	hosp_6m	Child admitted to hosptial/stay between 3 and 6 months of age
Infant Morbidities	Hospital visits	hospadmin_6m	How many hosptial admissions/stays between 3 and 6 months of age
Infant Morbidities	Hospital visits	hospadmin_cold_6m	Child hospital stay, between 3 and 6 months - Bad cold

Infant Morbidities	Hospital visits	hospadmin_fever_6m	Child hospital stay, between 3 and 6 months - Fever
Infant Morbidities	Hospital visits	hospadmin_rash_6m	Child hospital stay, between 3 and 6 months - Rash
Infant Morbidities	Hospital visits	hospadmin_wz_6m	Child hospital stay, between 3 and 6 months - Wheezing episode
Infant Morbidities	Hospital visits	hospadmin_ear_6m	Child hospital stay, between 3 and 6 months - Ear infection
Infant Morbidities	Hospital visits	hospadmin_allergy_6m	Child hospital stay, between 3 and 6 months - Allergy
Infant Morbidities	Hospital visits	hospadmin_asthma_6m	Child hospital stay, between 3 and 6 monthsh - Asthma
Infant Morbidities	Hospital visits	hospadmin_chest_6m	Child hospital stay, between 3 and 6 months - Chest
Infant Morbidities	Hospital visits	hospadmin_acci_6m	Child hospital stay, between 3 and 6 months - Accident
Infant Morbidities	Hospital visits	hospadmin_other_6m	Child hospital stay, between 3 and 6 months - Coughing
Infant Morbidities	Hospital visits	hospadmin_cough_6m	Child hospital stay, between 3 and 6 months - Other
Infant Morbidities	Hospital visits	hospadmin_otherspec_6m	Child hospital stay, between 3 and 6 months - Other Specify
Infant Morbidities	Hospital visits	hospstay_long_6m	Child longest hospital stay, between 3 and 6 months
Infant Morbidities	Hospital visits	hospstay_intubation_6m	Child hospital procedure, between 3 and 6 months - Intubation
Infant Morbidities	Hospital visits	hospstay_bloodtests_6m	Child hospital procedure, between 3 and 6 months - Blood tests
Infant Morbidities	Hospital visits	hospstay_facepuffer_6m	Child hospital procedure, between 3 and 6 months - Facemask or puffer
Infant Morbidities	Hospital visits	hospstay_IV_6m	Child hospital procedure, between 3 and 6 months - IV
Infant Morbidities	Hospital visits	hospstay_oxygen_6m	Child hospital procedure, between 3 and 6 months - Oxygen
Infant Morbidities	Hospital visits	hospstay_other_6m	Child hospital procedure, between 3 and 6 months - Other
Infant Morbidities	Hospital visits	hospstay_otherspec_6m	Child hospital procedure, between 3 and 6 months - Other, specify
Infant Morbidities	Hospital visits	hosp_ICU_6m	Child in ICU, between 3 and 6 months
Infant Morbidities	Hospital visits	hosp_coldcoughwz_6m	Child hospital stay associated with colds, cough, or wheezing; between 3 and 6 months
Infant Morbidities	Hospital visits	hosp_coldcoughwz_numstay_6m	Number of hosptial stayd for child associated with colds, cough, or wheezing; between 3 and 6 months
Infant Morbidities	Hospital visits	hosp_1y	Child admitted to hosptial/stay, in last 6 months
Infant Morbidities	Hospital visits	hospadmin_1y	How many hosptial admissions/stays in last 6 months
Infant Morbidities	Hospital visits	hospadmin_cold_1y	Child hospital stay, in last 6 months - Bad cold
Infant Morbidities	Hospital visits	hospadmin_fever_1y	Child hospital stay, in last 6 months - Fever
Infant Morbidities	Hospital visits	hospadmin_rash_1y	Child hospital stay, in last 6 months - Rash
Infant Morbidities	Hospital visits	hospadmin_wz_1y	Child hospital stay, in last 6 months - Wheezing episode
Infant Morbidities	Hospital visits	hospadmin_ear_1y	Child hospital stay, in last 6 months - Ear infection
Infant Morbidities	Hospital visits	hospadmin_allergy_1y	Child hospital stay, in last 6 months - Allergy
Infant Morbidities	Hospital visits	hospadmin_asthma_1y	Child hospital stay, in last 6 months - Asthma
Infant Morbidities	Hospital visits	hospadmin_chest_1y	Child hospital stay, in last 6 months - Chest
Infant Morbidities	Hospital visits	hospadmin_acci_1y	Child hospital stay, in last 6 months - Accident
Infant Morbidities	Hospital visits	hospadmin_other_1y	Child hospital stay, in last 6 months - Other
Infant Morbidities	Hospital visits	hospadmin_cough_1y	Child hospital stay, in last 6 months - Coughing

Infant Morbidities	Hospital visits	hospadmin_otherspec_1y	Child hospital stay, in last 6 months - Other Specify
Infant Morbidities	Hospital visits	hospstay_long_1y	Child longest hospital stay, in last 6 months
Infant Morbidities	Hospital visits	hospstay_intubation_1y	Child hospital procedure, in last 6 months - Intubation
Infant Morbidities	Hospital visits	hospstay_bloodtests_1y	Child hospital procedure, in last 6 months - Blood tests
Infant Morbidities	Hospital visits	hospstay_facepuffer_1y	Child hospital procedure, in last 6 months - Facemask or puffer
Infant Morbidities	Hospital visits	hospstay_IV_1y	Child hospital procedure, in last 6 months - IV
Infant Morbidities	Hospital visits	hospstay_oxygen_1y	Child hospital procedure, in last 6 months - Oxygen
Infant Morbidities	Hospital visits	hospstay_other_1y	Child hospital procedure, in last 6 months - Other
Infant Morbidities	Hospital visits	hospstay_otherspec_1y	Child hospital procedure, in last 6 months - Other, specify
Infant Morbidities	Hospital visits	hosp_ICU_1y	Child in ICU, in last 6 months
Infant Morbidities	Hospital visits	hosp_coldcoughwz_1y	Child hospital stay associated with colds, cough, or wheezing; in last 6 months
Infant Morbidities	Hospital visits	hosp_coldcoughwz_numstay_1y	Number of hosptial stayd for child associated with colds, cough, or wheezing; in last 6 months
Human Milk	HMOs	Mom_HMO_secretor	Mom_HMO_secretor
Human Milk	HMOs	Diversity_HMO	Diversity_HMO
Human Milk	HMOs	Evenness_HMO	Evenness_HMO
Human Milk	HMOs	2'FL_ug/mL	2’-fucosyllactose
Human Milk	HMOs	3FL_ug/mL	3-fucosyllactose
Human Milk	HMOs	LNnT_ug/mL	Lacto-N-neotetraose
Human Milk	HMOs	3'SL_ug/mL	3’-sialyllactose
Human Milk	HMOs	DFLac_ug/mL	Difucosyllactose
Human Milk	HMOs	6'SL_ug/mL	6’-sialyllactose
Human Milk	HMOs	LNT_ug/mL	Lacto-N-tetrose
Human Milk	HMOs	LNFP I_ug/mL	Lacto-N-fucopentaose-I
Human Milk	HMOs	LNFP II_ug/mL	Lacto-N-fucopentaose-II
Human Milk	HMOs	LNFP III_ug/mL	Lacto-N-fucopentaose-III
Human Milk	HMOs	LSTb_ug/mL	Sialyl-lacto-N-tetraose b
Human Milk	HMOs	LSTc_ug/mL	Sialyl-lacto-N-tetraose c
Human Milk	HMOs	DFLNT_ug/mL	Difucosyllacto-N-tetrose
Human Milk	HMOs	LNH_ug/mL	Lacto-N-hexaose
Human Milk	HMOs	DSLNT_ug/mL	Disialyllacto-N-tetraose
Human Milk	HMOs	FLNH_ug/mL	Fucosyllacto-N-hexaose
Human Milk	HMOs	DFLNH_ug/mL	Difucosyllacto-N-hexaose
Human Milk	HMOs	FDSLNH_ug/mL	Fucodisialyllacto-N-hexaose
Human Milk	HMOs	DSLNH_ug/mL	Disialyllacto-N-hexaose
Human Milk	HMOs	SUM_ug/mL	Summary measure to total HMO concentration (ug/ml)
Human Milk	HMOs	2'FL %	2’-fucosyllactose
Human Milk	HMOs	3FL %	3-fucosyllactose
Human Milk	HMOs	LNnT %	Lacto-N-neotetraose
Human Milk	HMOs	3'SL %	3’-sialyllactose
Human Milk	HMOs	DFLac %	Difucosyllactose
Human Milk	HMOs	6'SL %	6’-sialyllactose
Human Milk	HMOs	LNT %	Lacto-N-tetrose
Human Milk	HMOs	LNFP I %	Lacto-N-fucopentaose-I
Human Milk	HMOs	LNFP II %	Lacto-N-fucopentaose-II
Human Milk	HMOs	LNFP III %	Lacto-N-fucopentaose-III
Human Milk	HMOs	LSTb %	Sialyl-lacto-N-tetraose b
Human Milk	HMOs	LSTc %	Sialyl-lacto-N-tetraose c
Human Milk	HMOs	DFLNT %	Difucosyllacto-N-tetrose
Human Milk	HMOs	LNH %	Lacto-N-hexaose
Human Milk	HMOs	DSLNT %	Disialyllacto-N-tetraose
Human Milk	HMOs	FLNH %	Fucosyllacto-N-hexaose
Human Milk	HMOs	DFLNH %	Difucosyllacto-N-hexaose
Human Milk	HMOs	FDSLNH %	Fucodisialyllacto-N-hexaose

Human Milk	HMOs	DSLNH %	Disialyllacto-N-hexaose
Human Milk	HMOs	SUM %	Summary measure to total HMO concentration (%l)
Human Milk	Fatty Acids	C10:0 %	Capric acid
Human Milk	Fatty Acids	C12:0 %	Lauric acid
Human Milk	Fatty Acids	C14:0 %	Myristic acid
Human Milk	Fatty Acids	14:1n9 %	Tetradecanoic acid
Human Milk	Fatty Acids	C15:0 %	Pentadecanoic acid
Human Milk	Fatty Acids	C16:0 %	Palmitic acid
Human Milk	Fatty Acids	C16:1n9 %	Palmitoleic acid
Human Milk	Fatty Acids	C17:0 %	Margaric acid
Human Milk	Fatty Acids	C18:0 %	Stearic acid
Human Milk	Fatty Acids	TVA %	trans-vaccenic acid
Human Milk	Fatty Acids	C18:1n9 %	Oleic acid
Human Milk	Fatty Acids	C18:1C11 %	Vaccenic acid
Human Milk	Fatty Acids	C18:2n6 %	Linoleic acid (LA)
Human Milk	Fatty Acids	C20:0 %	Arachidic acid
Human Milk	Fatty Acids	C18:3n6 %	gamma-Linolenic acid (GLA)
Human Milk	Fatty Acids	C18:3n3 %	alpha-Linolenic acid (ALA)
Human Milk	Fatty Acids	C20:2n6 %	Eicosadienoic acid
Human Milk	Fatty Acids	C20:3n6 %	Dihomo- ɣ -linolenic acid (DGLA)
Human Milk	Fatty Acids	unk %	Unknown (Combine with AA?)
Human Milk	Fatty Acids	C20:4n6 %	Arachidonic acid (AA)
Human Milk	Fatty Acids	20:4n3 %	Eicosatetraenoic acid
Human Milk	Fatty Acids	CLA %	Conjugated linoleic acid
Human Milk	Fatty Acids	C20:5n3 %	Eicosapentaenoic acid (EPA)
Human Milk	Fatty Acids	C24:0 %	Tetracosanoic acid
Human Milk	Fatty Acids	C24:1n9 %	Nervonic acid
Human Milk	Fatty Acids	C22:4n6 %	Adrenic acid
Human Milk	Fatty Acids	22:5n6 %	Osbond acid
Human Milk	Fatty Acids	C22:5n3 %	Docosapentaenoic acid (DPA)
Human Milk	Fatty Acids	C22:6n3 %	Docosahexaenoic acid (DHA)
Human Milk	Fatty Acids	total lipid	Lipids weight in grams
Human Milk	Hormones	milk_adiponectin	Adiponectin (ng/ml)
Human Milk	Hormones	milk_insulin	Insulin (pg/mL)
Human Milk	Hormones	milk_leptin	Leptin (pg/mL)
Human Milk	Immunomodulators	milk_norm_Lactoferrin	Lactoferrin
Human Milk	Immunomodulators	milk_norm_CD14	Cluster of differentiation 14
Human Milk	Immunomodulators	milk_norm_IgE	Immunoglobulin E
Human Milk	Immunomodulators	milk_norm_IgG1	Immunoglobulin G1
Human Milk	Immunomodulators	milk_norm_IgG2	Immunoglobulin G2
Human Milk	Immunomodulators	milk_norm_IgG3	Immunoglobulin G3
Human Milk	Immunomodulators	milk_norm_IgG4	Immunoglobulin G4
Human Milk	Immunomodulators	milk_norm_IgM	Immunoglobulin M
Human Milk	Immunomodulators	milk_norm_GRO	C-X-C motif chemokine ligand 1 (CXCL1)
Human Milk	Immunomodulators	milk_norm_IL-8	Interleukin-8 (CXCL8)
Human Milk	Immunomodulators	milk_norm_MDC	Macrophage-derived (CCL22)
Human Milk	Immunomodulators	milk_norm_Fractakine	Fractakine (CX3CL1)
Human Milk	Immunomodulators	milk_norm_MCP-1	Monocyte chemoattractant protein 1 (CCL1)
Human Milk	Immunomodulators	milk_norm_IL-9	Interleukin-9
Human Milk	Immunomodulators	milk_norm_EGF	Epidermal growth factor
Human Milk	Immunomodulators	milk_norm_VEGF	Vascular endothelial growth factor
Human Milk	Immunomodulators	milk_norm_IP-10	Interferon-gamma-induced protein 10 (CXCL10)
Human Milk	Immunomodulators	milk_norm_CCL5	C-C motif chemokine 5 (RANTES)
Human Milk	Immunomodulators	milk_norm_G-CSF	Mammalian granulocyte colony-stimulating factor
Human Milk	Immunomodulators	milk_norm_IL-4	Interleukin-4
Human Milk	Immunomodulators	milk_norm_IL-6	Interleukin-6
Human Milk	Immunomodulators	milk_norm_TNF	Tumor necrosis factor
Human Milk	Immunomodulators	milk_norm_IL-33	Interleukin-33
Human Milk	Immunomodulators	milk_norm_TSLP	Thymic stromal lymphopoietin
Human Milk	Immunomodulators	milk_norm_TLR2	Soluble toll-like receptor 2
Human Milk	Immunomodulators	milk_norm_IgA	Immunoglobulin A

Human Milk	Immunomodulators	milk_norm_TGF-b2	Transforming growth factor β2
Human Milk	Immunomodulators	milk_norm_TGF-b1	Transforming growth factor β1
